# Associations of specific food sources of dietary fat with prostate cancer incidence and mortality: results from a large prospective cohort

**DOI:** 10.3389/fnut.2025.1630437

**Published:** 2025-11-19

**Authors:** Yong-xin Fu, Zi Ye, Ya-dong Li, Ning Liu, Xue-song Chen, Xiao-liang Jiang, Ke Li

**Affiliations:** 1 Department of Urology, The Banan Affiliated Hospital of Chongqing Medical University, Chongqing, China; 2 Department of Gastroenterology, Beijing Friendship Hospital, Capital Medical University, Beijing, China; 3 Department of Urology, The Second Affiliated Hospital of Chongqing Medical University, Chongqing, China

**Keywords:** fat and fatty acids, prostate cancer (PCa), prospective cohort, cancer prevention, risk factor

## Abstract

**Background:**

Although fat intake has been implicated in prostate cancer (PCa) risk, the specific impact of dietary fat from specific food sources on PCa susceptibility in United States populations remains unclear.

**Methods:**

This prospective cohort included 49,424 men from the Prostate, Lung, Colorectal, and Ovarian Cancer Screening Trial. Cox proportional hazards regression was used to evaluate the risk of PCa incidence and mortality. Subgroup and sensitivity analyses were performed to investigate the potential effect modifiers.

**Results:**

During follow-up, we documented 4,308 incident cases of PCa, of whom 392 died from PCa. Total amount and specific types of fat intakes were not associated with PCa incidence and mortality. When considering available food sources, a greater intake of fat from dairy (HR _Q4vs.Q1_:1.13; 95% CI: 1.02–1.25; *P*_trend_ = 0.069) and saturated fatty acids (SFAs) from dairy (HR _Q4vs.Q1_:1.12; 95% CI: 1.01–1.24; *P*_trend_ = 0.059) was associated with a higher incidence of PCa in a linear dose-response association (all *P*_non − linearity_ >0.05). However, a greater intake of plant-based monounsaturated fatty acids (MUFAs; HR _Q4vs.Q1_: 0.67; 95% CI: 0.48–0.94; *P*_trend_ = 0.023), plant-based SFAs (HR _Q4vs.Q1_:0.65; 95% CI: 0.47–0.91; *P*_trend_ = 0.026) and polyunsaturated fatty acids (PUFAs) from fish (HR _Q4vs.Q1_: 0.48; 95% CI: 0.48–0.87; *P*_trend_ = 0.005) was associated with a decreased PCa mortality in a non-linear dose-response relationships (all *P*_non − linearity_ < 0.05). The reliability of these results was supported by sensitivity and subgroup analyses.

**Conclusion:**

These findings demonstrate that the specific food sources of fat rather than total amount were significantly associated with PCa incidence and mortality.

## 1 Introduction

Prostate cancer (PCa) is the most common solid malignancy in men, accounting for 299,010 new cancer diagnoses and 35,250 deaths in the United States, with a continuously increasing burden of disease (1). Age, ethnicity, and family history are well-established risk factors for PCa (2–4). However, incidence and mortality rates of PCa vary substantially across different countries and geographic locations, with the highest incidence rates observed in Northern and Western Europe, and the highest mortality rates observed in the Caribbean and African countries (5), indicating some modifiable risk factors should be considered vitally crucial to the primary prevention of PCa. Although the specific mechanisms of PCa remain poorly understood, some epidemiological evidence showed that unbalanced and harmful dietary changes may be potential risk factors for the development of overall and aggressive PCa (6–8).

Dietary fats are critical macronutrients that have essential biological functions including energy storage, acting as a signaling molecule, serving as a structural component of membranes, and transport and absorption of fat-soluble vitamins (9). Some animal studies have repeatedly supported the involvement of fat or fatty acids in prostate tumorigenesis and progression (10, 11). For example, extrinsic saturated fat intake promoted PCa mortality by enriching for a Myc proto-oncogene-transcriptional program or synergies with Myc proto-oncogene over expression, which is observed in 37% of metastatic prostate. However, epidemiological research has not shown any clear associations with this malignancy (12). The European Association of Urology Guidelines on PCa consider red meat, total meat, and processed meat consumption as possible risk factors, whereas long-chain omega-3 polyunsaturated fatty acids (PUFAs) no been suggested as risk or protective factors due to a lack of established associations (13). Recently, some evidence further indicated that the impact of dietary fat on potential health outcomes appears to depend on the fat quality and sources (i.e., animal-derived or plant-derived) (14–17). For example, a prospective cohort study demonstrated that animal-based fats were associated with elevated risks of overall and cardiovascular disease mortality, and the intake of plant fat was inverse associations (14).

Although the health effects of diverse dietary fats depend on available food sources, there are no prospective data elucidating the associations between fat quality and specific food sources and PCa incidence and mortality. To provide effective strategic guidance for dietary prevention, we comprehensively evaluated the associations of dietary fat from various food sources with the PCa incidence and mortality in the Prostate, Lung, Colorectal, and Ovarian (PLCO) Cancer Screening Cohort.

## 2 Methods

### 2.1 Study population

The PLCO Cancer Screening Trial, which was a randomized multicenter controlled study, was aimed to determine whether screening exams or tests could reduce the risk of mortality from prostate, lung, colorectal and ovarian cancers. Study design and methodology of the PLCO trial have been reported in detail elsewhere (18). Briefly, participants were aged 55–74 years in this trial during the period from 1993 to 2001 from 10 participating screening centers (St Louis, Denver, Detroit, Salt Lake City, Minneapolis, Marshfield, Birmingham, Pittsburgh, Washington, and Honolulu). Based on the predefined eligible criteria, approximately 155,000 individuals were enrolled and randomly assigned to the screening arm and control arm in equal proportions (Supplementary Figure S1). Individuals in control arm received the usual care, whereas those in screening arm received a cancer screening intervention, including prostate-specific antigen testing and digital rectal exams for PCa screening, posteroanterior chest X-ray to screen lung cancer, and the flexible sigmoidoscopy to screen colorectal cancer. The PLCO trial was approved by the United States National Cancer Institute and the Institutional Review Board of each screening center. All of the participants provided their informed consents.

**Figure 1:**
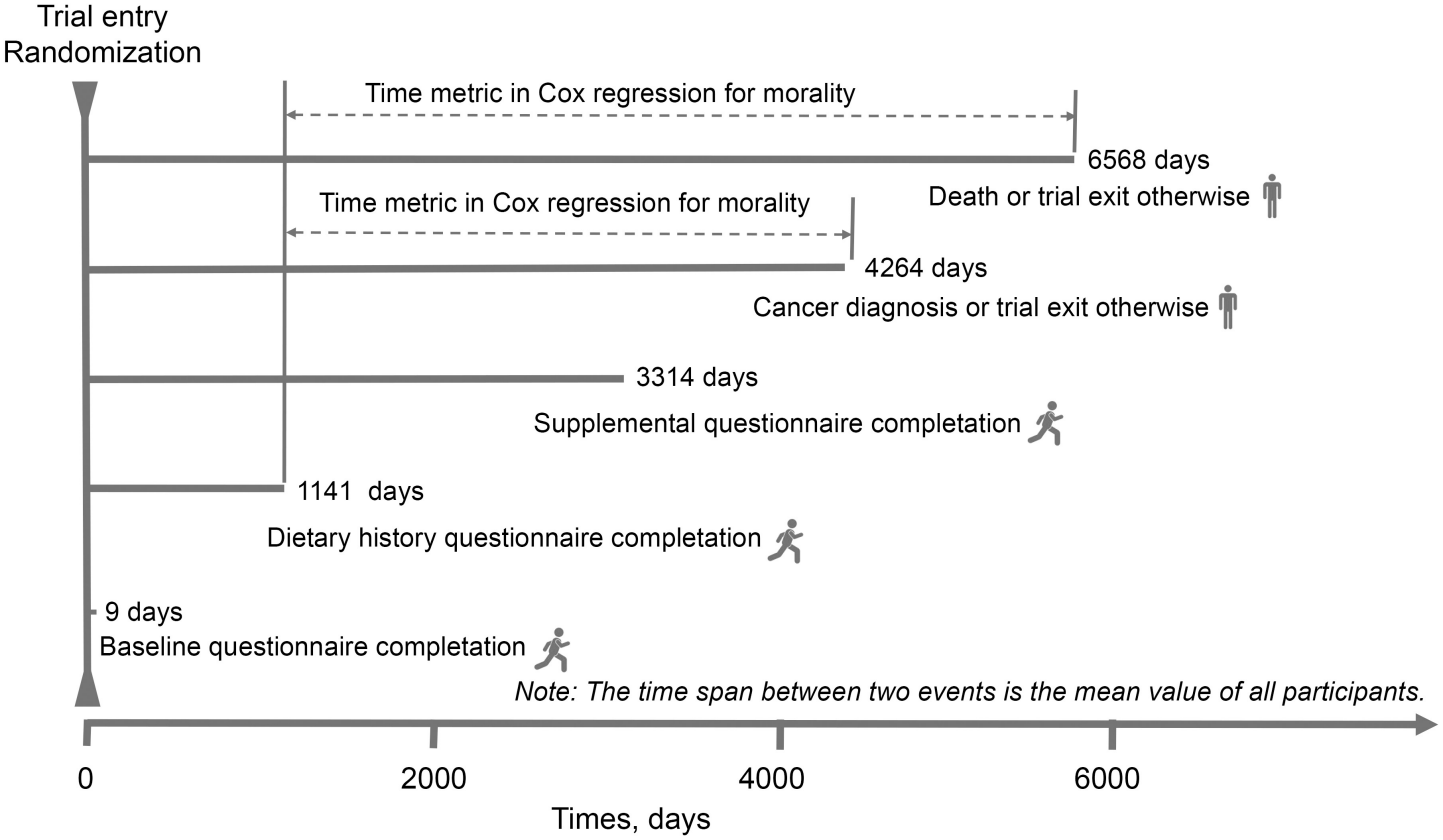
The timeline and follow-up scheme of our study. The baseline point in this study was set at the date of diet history questionnaire completion.

In this study, due to the fact that the outcome was PCa, the considering study population was only men. The following males were further excluded: (1) overall 2,824 males without returning a baseline questionnaire; (2) overall 17,510 males without completing a Dietary History Questionnaire (DHQ); (3) overall 2,622 with providing an invalid DHQ. A valid DHQ referred to the presence of having a DHQ completion date, DHQ completion date before death date, < 8 missing DHQ items, and the absence of extreme values of calorie intake (lowest or highest 1%). Notably, the above-mentioned criteria were jointly defined by nutritionists, epidemiologists, and statisticians from the United States National Cancer Institute; (4) Overall 4,194 males diagnosed with cancer before DHQ completion; (5) those with PCa diagnosed or dead or without annual study update ≤ 1 year after study entry (*n* = 97); and (6) overall seven males with outcome events observed between trial entry and DHQ completion (outcome events referred to loss to follow-up, death or incident PCa). After these exclusions, a total of 49,424 male participants were included in this cohort. The flowchart is in Figure 1. Noteworthy, after comparing the populations between inclusion and exclusion, the standardized differences were found to be < 0.1, which indicated that the possibility of non-participation bias was small because of the exclusion of numerous male participants (Supplementary Table S1).

### 2.2 Outcome ascertainment

In this PLCO Cancer Screening Trial, PCa was ascertained mainly via an annual study update form that was mailed to all study participants to report whether the enrolled participants received the diagnosis of PCa, the date and location of diagnosis, as well as contact information of their physicians. A standardized form was used to extract relevant medical records for further confirming the diagnosis, clinical stage and grade. Fatal PCa was defined as PCa–specific mortality (PCa was the underlying cause of death) (19).

### 2.3 Data collection

A baseline questionnaire solicited information on age, race, education, physical activity, smoking status, family history of PCa and history of diabetes, and other factors. DHQ, a food frequency questionnaire including the portion size and frequency of intake of 124 food items and supplement use during the past year, was used to collect dietary information. Age at DHQ completion and alcohol intake were collected through this questionnaire. The amount of daily food consumption was estimated by multiplying food frequency by portion size; the amount of daily energy and nutrient intake was calculated by the detailed analysis file of DietCalc (National Cancer Institute, Bethesda, MD), which determined frequency of consumption and serving size question and used nutrient values based on national dietary data. Healthy Eating Index-2015 was index reflecting an individual's diet quality and was calculated as stated previously. Physical activity level referred to the total time of moderate-to-vigorous activity per week, which was evaluated by a self-administered supplemental questionnaire.

Before conducting data analyses, we used the residual method to adjust fat and fatty acids consumption to energy intake from diet for minimizing the extraneous variation of fat and fatty acids consumption because of energy intake (20).

### 2.4 Statistical analysis

For all covariates except physical activity, 5% or less of values were missing and were imputed to the modal value (for categorical variables) or median (for continuous variables). For physical activity, the proportion of missing values was higher (27.52%) and these values were considered as missing at random and then multiple imputation with chained equations was used to impute them (the number of imputations set at 25). A missing data was included into the models for this variable because massive imputation for a non-negligible number of participants and risk of selection bias was considered. We further conduct main analyses in participants with complete data for comparison (Supplementary Table S2). The differences in participants' baseline characteristics between quarters of the fat and fatty acids were examined by using analysis of variance or χ^2^ tests wherever appropriate. Follow-up started from the date of DHQ completion until to the date of PCa diagnosis/death (event), or 31 December 2009, whichever occurred first. For the follow-up time of PCa mortality, the end of mortality follow-up was 2018, which was detailed on the PLCO website (https://cdas.cancer.gov/learn/plco/early-qx/; Figure 1). We conducted Cox proportional hazards regression, with person-time as the underlying time metric to compute hazard ratios (HRs) and 95% confidence intervals (CIs) for the association between dietary fat and fat acids intake and PCa or fatal PCa risk. Covariates were selected on the basis of those from literature previous literature to have an association with the risk of PCa. Model 1 was adjusted for age at DHQ completion and race/ethnicity; model 2 was additionally adjusted for established variables for PCa incidence including body mass index (BMI, continuous), alcohol consumption (g/day, continuous), smoking status, education, energy intake (kcal/day, continuous), family history of PCa, aspirin use and history of diabetes. To test for the potential influence of the nutritional quality of the diet in this association, model 3 was additionally adjusted for total protein, carbohydrates, fiber, and sodium.

Subgroup analyses were conducted to determine whether the observed associations between ultra-processed food consumption and risk of PCa were modified by age at DHQ completion (>65 vs. < 65 years), BMI (>25 vs. < 25), smoking status (current or former vs. never), trial group (screening compared with control groups), and alcohol consumption (≥median vs < median). A *P* value for interaction was obtained by comparing models with and without interaction terms before performing the above-mentioned subgroup analyses to avert the possibly spurious subgroup differences.

Restricted cubic spline regression with three knots (i.e., 10th, 50th, and 90th percentiles) was used to accurately describe the association between ultra-processed food consumption and risk of PCa, using 0 servings/day as the reference level. It is worth noting that number of knots was ascertained according to the Akaike's information criterion and the Bayesian information criterion, with the lowest values representing the best-fitted model. A *P* value for non-linearity was obtained by examining the hypothesis that the regression coefficient of the second spline was equal to 0.

We did sensitivity analyses based on model 3 by excluding PCa cases diagnosed within the first 2, 3, or 5 years of follow-up to avoid reverse causality bias, and excluding individuals with extreme energy intake (top 2.5% or bottom 2.5%). To alleviate residual confounding, we additional adjusted for (1) smoking status, marital status, aspirin use, education on model 2, (2) physical activity level on model 2, (3) physical activity, overall fruit, vegetable, red and processed meat and coffee consumption on model 2. All statistical analyses were performed using R software version 4.3.1. Two-sided *P* < 0.05 was considered statistically significant.

## 3 Results

### 3.1 Participant characteristics

In the whole study population, a total of 49,424 participants [mean (SD) age, 65.8 (5.7) years] were included. Baseline characteristics of PLCO participants, stratified by total dietary fat or specific fat types [including monounsaturated fatty acids (MUFAs), polyunsaturated fatty acids (PUFAs), saturated fatty acids (SFAs), and total fatty acids (TFAs)], are reported in Table 1 and Supplementary Tables S3–S6, respectively. Participants with a greater intake of dietary fat were more likely to have diabetes, higher BMI, and lower intake of dietary fiber, whole grain, alcohol, and fruits and vegetables. These participants have lower physical activity levels, and Healthy Eating Index-2015 but have greater intakes of total energy, meat, carbohydrates, protein, cholesterol and sodium, and distributions of baseline participant characteristics by specific types of fat were similar to those according to quartile of dietary fat (Supplementary Tables S3–S6).

**Table 1:** Baseline characteristics of the PLCO study population according to quarters of dietary fat intake (*n* = 49,424).

**Characteristics**	**Quarters of total fat intake** ^*^					***P* for trend ^a^**
	**All participants**	**Q1 (*****n*** = **12,356)**	**Q2 (*****n*** = **12,356)**	**Q3 (*****n*** = **12,356)**	**Q4 (*****n*** = **12,356)**	
Age, years	65.8 (5.7)	66.1 (5.8)	66.3 (5.8)	65.8 (5.8)	64.9 (5.5)	< 0.001
Body mass index (kg/m^2^)	27.5 (4.1)	26.8 (3.7)	27.3 (3.9)	27.6 (4.0)	28.3 (4.4)	< 0.001
Physical activity (min/week)^b^	134.7 (129.1)	148.9 (135.2)	135.0 (127.3)	128.1 (125.6)	126.5 (126.9)	< 0.001
Family history of prostate cancer, *n* (%)	3,591 (7.3%)	894 (7.2%)	904 (7.3%)	870 (7.0%)	923 (7.5%)	0.119
History of diabetes, *n* (%)	3,949 (8.0%)	698 (5.6%)	970 (7.9%)	1,048 (8.5%)	1,233 (10.0%)	< 0.001
Aspirin user, *n* (%)	25,606 (52.2%)	6,768 (55.1%)	6,396 (52.1%)	6,247 (50.9%)	6,195 (50.5%)	< 0.001
**Racial/ethnic group**, ***n*** **(%)**						
Non-Hispanic White	44,846 (90.7%)	11,013 (89.1%)	11,138 (90.1%)	11,277 (91.3%)	11,418 (92.4%)	< 0.001
Non-Hispanic Black	1,341 (2.7%)	333 (2.7%)	324 (2.6%)	339 (2.7%)	345 (2.8%)	
Hispanic	864 (1.7%)	165 (1.3%)	226 (1.8%)	228 (1.8%)	245 (2.0%)	
Other race/ethnicity^c^	2,373 (4.8%)	845 (6.8%)	668 (5.4%)	512 (4.1%)	348 (2.8%)	
**Educational degree**, ***n*** **(%)**						
Postgraduate	11,070 (22.4%)	3,376 (27.3%)	2,958 (23.9%)	2,509 (20.3%)	2,227 (18.0%)	< 0.001
College graduate	9,654 (19.5%)	2,586 (20.9%)	2,503 (20.3%)	2,369 (19.2%)	2,196 (17.8%)	
College below	28,700 (58.1%)	6,394 (51.7%)	6,895 (55.8%)	7,478 (60.5%)	7,933 (64.2%)	
**Smoking status**, ***n*** **(%)**						
Never	18,592 (37.6%)	4,833 (39.1%)	4,931 (39.9%)	4,651 (37.6%)	4,177 (33.8%)	< 0.001
Current	4,987 (10.1%)	928 (7.5%)	962 (7.8%)	1,304 (10.6%)	1,793 (14.5%)	
Former	25,845 (52.3%)	6,595 (53.4%)	6,463 (52.3%)	6,401 (51.8%)	6,386 (51.7%)	
Alcohol intake, g/d	14.3 (33.6)	30.8 (59.6)	10.5 (16.2)	8.3 (13.2)	7.6 (12.7)	< 0.001
Energy intake from diet, kcal/day	1,994.5 (814.5)	2,125.8 (833.1)	1,726.9 (677.3)	1,774.5 (702.8)	2,350.7 (861.8)	< 0.001
Healthy Eating Index-2015	66.5 (9.7)	69.7 (8.9)	67.2 (8.6)	63.1 (8.9)	58.6 (9.0)	< 0.001
**Food consumption**						
Whole grain (g/day)	66.1 (66.0)	89.5 (84.8)	68.4 (61.8)	55.2 (52.4)	51.4 (53.0)	< 0.001
Vegetable (g/day)	290.0 (193.1)	338.8 (233.4)	265.9 (172.0)	253.4 (165.7)	302.0 (182.2)	< 0.001
Fruit (g/day)	265.6 (223.1)	384.5 (306.9)	267.6 (177.2)	214.3 (161.3)	196.1 (161.0)	< 0.001
Red meats, processed (g/day)	17.1 (18.6)	11.6 (13.7)	13.0 (13.6)	16.2 (14.7)	27.5 (25.3)	< 0.001
Red meats, not processed (g/day)	62.2 (50.8)	46.8 (39.3)	50.4 (37.9)	59.8 (42.4)	91.7 (65.4)	< 0.001
White meats (g/day)	52.0 (49.3)	56.8 (53.1)	46.3 (43.3)	46.1 (43.0)	58.6 (55.4)	< 0.001
**Nutrient intake**						
SAFAs (g/day)	23.4 (13.1)	17.3 (8.9)	18.5 (9.1)	22.3 (9.9)	35.4 (15.0)	< 0.001
PUFAs (g/day)	15.7 (8.3)	12.6 (6.1)	12.7 (6.0)	14.7 (6.4)	22.9 (9.5)	< 0.001
MUFAs (g/day)	27.7 (14.7)	20.9 (10.3)	22.0 (10.3)	26.3 (10.8)	41.7 (16.2)	< 0.001
Carbohydrate (g/day)	245.7 (98.9)	291.0 (105.7)	227.2 (85.0)	215.3 (87.2)	249.2 (99.1)	< 0.001
TFAs (g/day)	4.7 (2.7)	3.7 (2.0)	3.9 (2.1)	4.5 (2.2)	6.7 (3.1)	< 0.001
Protein (g/day)	75.9 (33.6)	75.3 (30.7)	66.2 (28.5)	68.9 (29.5)	93.2 (38.0)	< 0.001
Cholesterol (mg/day)	247.8 (151.8)	189.4 (110.9)	198.6 (110.4)	236.8 (119.2)	366.2 (182.9)	< 0.001
Dietary fiber (g/day)	19.2 (9.1)	23.2 (10.4)	17.8 (7.7)	16.5 (7.7)	19.4 (8.7)	< 0.001
Sodium (mg/day)	3,121.0 (1,340.6)	3,079.9 (1,250.8)	2,745.1 (1,162.5)	2,853.8 (1,189.3)	3,805.2 (1,475.4)	< 0.001
**Body mass index**, ***n*** **(%)**						
Underweight (< 18.5 kg/m^2^)	131 (0.3%)	34 (0.3%)	30 (0.2%)	37 (0.3%)	30 (0.2%)	< 0.001
Normal (18.5–24.9 kg/m^2^)	12,733 (25.8%)	3,956 (32.0%)	3,294 (26.7%)	2,960 (24.0%)	2,523 (20.4%)	
Overweight (25–29.9 kg/m^2^)	25,614 (51.8%)	6,339 (51.3%)	6,515 (52.7%)	6,528 (52.8%)	6,232 (50.4%)	
Obese (>30 kg/m^2^)	10,946 (22.1%)	2,027 (16.4%)	2,517 (20.4%)	2,831 (22.9%)	3,571 (28.9%)	

^*^Values are mean ± SD or numbers (percentages).

^a^P value for comparison between quarters of total fat intake, by Kruskal-Wallis rank sum test or pearson's Chi-squared test.

^b^Total time of moderate-to-vigorous physical activity per week.

^c^Other race/ethnicity = Asian, Pacific Islander or American Indian.

### 3.2 Association between specific sources of fat and PCa incidence

**Figure 2:**
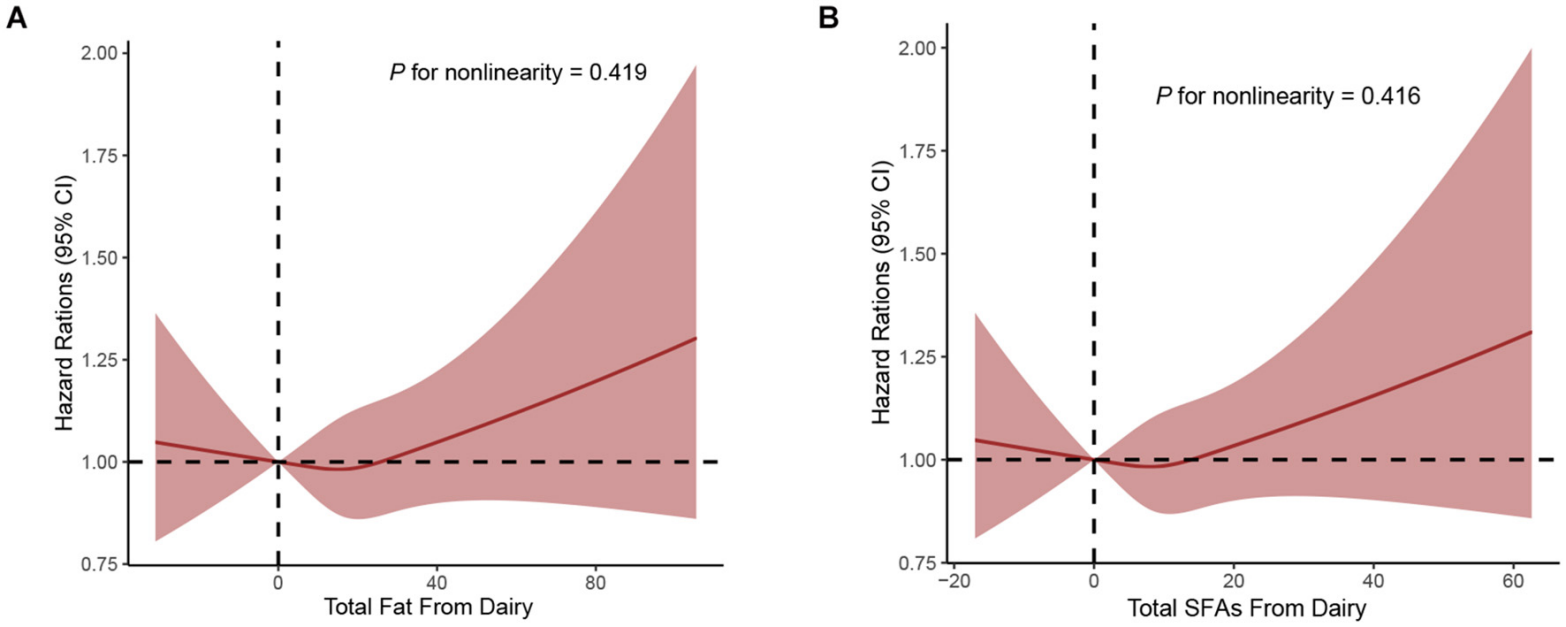
Dose-response analyses on the associations of total fat from dairy **(A)**, SFAs from dairy **(B)** with the incidence of PCa.

During a median follow-up of 9.26 years, a total of 4,308 incident cases of PCa, the overall incidence rate of PCa in this study population was 104.19 cases per 10,000 person-years. As Supplementary Table S7 shows, after full adjustment for potential confounders including demographic characteristics and key dietary nutrients (model 3), higher dietary total fat, SFAs, MUFAs, PUFAs, and trans-fat acids intake were not associated with the incidence of PCa, with the HRs _Q4vs.Q1_ (95%CIs) for overall PCa incidence being 1.09 (0.93–1.28), 0.95 (0.82–1.10), 1.03 (0.92–1.15), 1.12 (0.98–1.27), and 0.95 (0.86–1.06), respectively (all *P* for trend >0.05). Intriguingly, when considering fat and fatty acid sources, we observed a positive association of total fat from dairy with the incidence of PCa (full adjusted HR _Q4vs.Q1_: 1.13; 95% CI: 1.02–1.25; *P* for trend = 0.069), and association similar to PCa incidence was observed between SFAs from dairy intake (full adjusted HR _Q4vs.Q1_: 1.12; 95% CI: 1.01–1.25; *P* for trend = 0.059) in the Table 2. By using the restricted cubic spline regression, there was no evidence for non-linear associations of dietary SFAs from dairy or total fat from dairy intake with the incidence of PCa (all *P* non-linearity >0.05; Figure 2).

**Table 2:** Association between dietary fat and fatty acids by dietary sources and the incidence of PCa^a^.

**Dietary sources**	**Quartile 1**	**Quartile 2**	**Quartile 3**	**Quartile 4**	***P-*trend**
**Total fat from**					
Animals^b^	1.00 (reference)	1.03 (0.95–1.14)	1.02 (0.92–1.13)	1.06 (0.94–1.20)	0.407
Dairy	1.00 (reference)	1.03 (0.94–1.13)	0.97 (0.88–1.07)	**1.13 (1.02–1.25)**	0.069
Egg	1.00 (reference)	1.05 (0.96–1.15)	1.00 (0.91–1.10)	1.05 (0.95–1.15)	0.589
Fish	1.00 (reference)	0.98 (0.90–1.08)	0.98 (0.89–1.08)	0.93 (0.85–1.02)	0.137
Meat	1.00 (reference)	1.05 (0.96–1.15)	1.04 (0.95–1.15)	1.02 (0.91–1.14)	0.138
Plants	1.00 (reference)	1.03 (0.94–1.12)	1.01 (0.92–1.11)	0.98 (0.88–1.09)	0.702
**MUFAs from**					
Animals^b^	1.00 (reference)	1.06 (0.97–1.16)	1.00 (0.90–1.10)	1.06 (0.94–1.20)	0.540
Dairy	1.00 (reference)	1.05 (0.95–1.15)	0.98 (0.89–1.08)	1.08 (0.98–1.20)	0.308
Egg	1.00 (reference)	1.00 (0.92–1.10)	0.99 (0.90–1.09)	0.98 (0.89–1.09)	0.692
Fish	1.00 (reference)	1.10 (1.01–1.19)	1.01 (0.93–1.10)	0.93 (0.84–1.02)	0.045
Meat	1.00 (reference)	1.07 (0.98–1.17)	1.04 (0.94–1.15)	1.06 (0.95–1.18)	0.444
Plants	1.00 (reference)	1.00 (0.91–1.09)	0.94 (0.85–1.03)	0.93 (0.84–1.03)	0.079
**PUFAs from**					
Animals^b^	1.00 (reference)	1.00 (0.91–1.09)	1.00 (0.91–1.10)	0.99 (0.89–1.10)	0.871
Dairy	1.00 (reference)	1.05 (0.96–1.15)	1.06 (0.96–1.16)	1.08 (0.98–1.20)	0.132
Egg	1.00 (reference)	1.08 (0.98–1.18)	1.03 (0.93–1.14)	1.03 (0.93–1.14)	0.832
Fish	1.00 (reference)	1.03 (0.95–1.12)	0.97 (0.89–1.06)	0.92 (0.84–1.01)	0.043
Meat	1.00 (reference)	1.02 (0.93–1.11)	1.05 (0.95–1.15)	1.01 (0.91–1.12)	0.709
Plants	1.00 (reference)	1.01 (0.92–1.10)	1.00 (0.90–1.09)	1.03 (0.93–1.14)	0.662
**SFAs from**					
Animals^b^	1.00 (reference)	1.02 (0.93–1.12)	1.03 (0.93–1.14)	1.10 (0.98–1.24)	0.103
Dairy	1.00 (reference)	1.02 (0.93–1.12)	1.00 (0.90–1.10)	**1.12 (1.01–1.24)**	0.059
Egg	1.00 (reference)	1.03 (0.95–1.12)	0.94 (0.86–1.03)	0.97 (0.88–1.07)	0.268
Fish	1.00 (reference)	1.04 (0.96–1.14)	1.00 (0.92–1.09)	0.91 (0.83–1.01)	0.047
Meat	1.00 (reference)	1.05 (0.96–1.15)	1.05 (0.95–1.15)	1.04 (0.93–1.16)	0.535
Plants	1.00 (reference)	0.98 (0.90–1.08)	1.00 (0.91–1.10)	0.92 (0.83–1.03)	0.185

^a^Values are hazard ratios (95% confidence intervals).

^b^Animal sources include contributions from meat, dairy, eggs, and fish.

Hazard ratios in bold represent statistically significant associations.

### 3.3 Association between specific sources of fat and PCa mortality

A total of 392 PCa deaths were recorded during up to 24 years of follow-up, covering 725,377 person-years, with the overall incidence rate of 5.40 cases per 10,000 person-years. Higher intake of total fat, SFAs, MUFAs, PUFAs, and trans-fat acids were not significantly associated with the risk of fatal PCa (quartiles 4 vs 1: fully adjusted HR: 1.11, 95%CI: 0.65–1.88; HR: 0.86, 95%CI: 0.53–1.40; HR: 0.79, 95%CI: 0.55–1.13; HR: 1.32, 95%CI: 0.85–2.06, HR: 0.92, 95%CI: 0.66–1.28, respectively; all *P*-trend >0.05; Supplementary Table S8).

**Figure 3:**
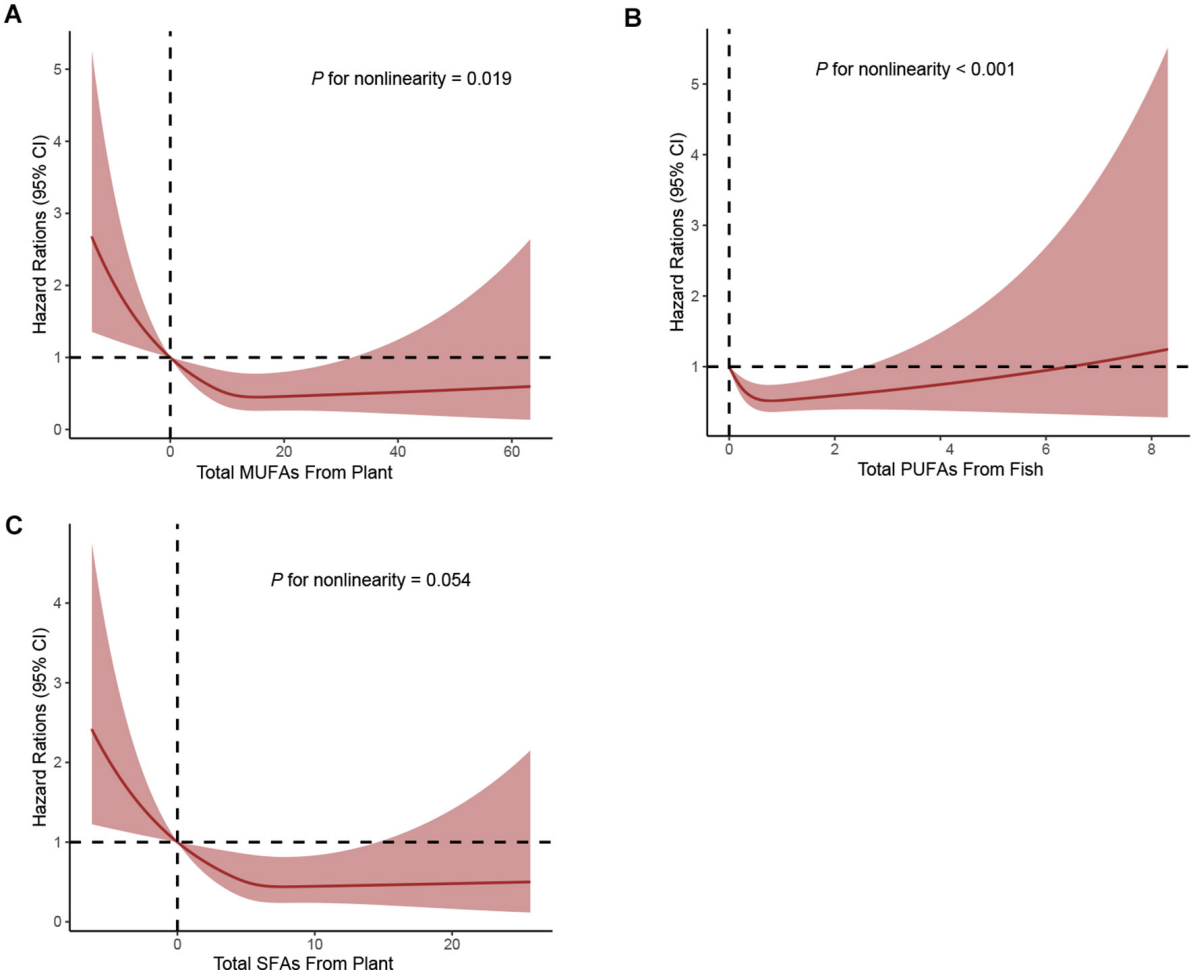
Dose-response analyses on the associations of MUFAs from plant **(A)**, PUFAs from fish **(B)**, SFAs from plant **(C)** with the mortality of PCa.

When considering the sources of dietary fat and PCa mortality, significant inverse associations with PCa mortality were observed between MUFAs from plants (full adjusted HR _Q4vs.Q1_: 0.67; 95% CI: 0.48–0.94; *P* for trend = 0.023) and SFAs from plants (full adjusted HR _Q4vs.Q1_: 0.65; 95% CI: 0.47–0.91; *P* for trend = 0.026) in the Table 3. In addition, greater intake of PUFAs from fish was associated with a lower mortality of PCa (full adjusted HR _Q4vs.Q1_: 0.65; 95% CI: 0.48–0.87; *P* for trend = 0.005) in the Table 3. Finally, using restricted cubic spline regression, we observed non-linear inverse dose-response associations of MUFAs from plants (*P* non-linearity = 0.019), SFAs from plants (*P* non-linearity = 0.054) and PUFAs from fish (*P* non-linearity < 0.001) with PCa mortality (Figures 3A–C).

**Table 3:** Association between dietary fat and fatty acids by intake source and the morality of PCa^a^.

**Dietary sources**	**Quartile 1**	**Quartile 2**	**Quartile 3**	**Quartile 4**	***P-*trend**
**Total fat from** ^b^					
Animals	1.00 (reference)	1.11 (0.80–1.53)	1.27 (0.91–1.79)	1.22 (0.82–1.82)	0.261
Dairy	1.00 (reference)	0.87 (0.63–1.20)	0.84 (0.60–1.18)	1.19 (0.85–1.66)	0.375
Egg	1.00 (reference)	0.87 (0.64–1.19)	0.97 (0.71–1.32)	1.09 (0.81–1.48)	0.448
Fish	1.00 (reference)	0.79 (0.59–1.06)	0.67 (0.49–0.92)	0.79 (0.59–1.06)	0.072
Meat	1.00 (reference)	1.01 (0.75–1.36)	0.92 (0.66–1.27)	0.99 (0.69–1.41)	0.829
Plants	1.00 (reference)	0.85 (0.63–1.13)	0.68 (0.50–0.93)	0.76 (0.54–1.07)	0.062
**MUFAs from** ^b^					
Animals	1.00 (reference)	1.27 (0.93–1.74)	1.06 (0.75–1.51)	1.29 (0.87–1.92)	0.363
Dairy	1.00 (reference)	0.84 (0.62–1.14)	0.74 (0.53–1.03)	1.01 (0.72–1.41)	0.846
Egg	1.00 (reference)	0.74 (0.54–1.01)	0.85 (0.61–1.17)	0.94 (0.67–1.29)	0.927
Fish	1.00 (reference)	0.70 (0.54–0.93)	0.59 (0.45–0.80)	0.70 (0.52–0.95)	0.014
Meat	1.00 (reference)	1.06 (0.79–1.44)	1.02 (0.74–1.41)	1.03 (0.72–1.48)	0.931
Plants	1.00 (reference)	**0.74 (0.56–1.00)**	**0.71 (0.52–0.95)**	**0.67 (0.48–0.94)**	**0.023**
**PUFAs from** ^b^					
Animals	1.00 (reference)	0.96 (0.71–1.29)	0.75 (0.54–1.04)	1.00 (0.72–1.40)	0.668
Dairy	1.00 (reference)	0.67 (0.49–0.93)	1.00 (0.74–1.36)	0.73 (0.52–1.01)	0.296
Egg	1.00 (reference)	0.77 (0.57–1.05)	0.81 (0.59–1.14)	0.90 (0.65–1.24)	0.611
Fish	1.00 (reference)	**0.59 (0.45–0.78)**	**0.56 (0.42–0.74)**	**0.65 (0.48–0.87)**	**0.005**
Meat	1.00 (reference)	1.02 (0.76–1.36)	0.85 (0.63–1.17)	1.10 (0.79–1.55)	0.822
Plants	1.00 (reference)	0.80 (0.60–1.07)	0.67 (0.49–0.91)	0.74 (0.53–1.04)	0.048
**SFAs from** ^b^					
Animals	1.00 (reference)	1.20 (0.87–1.66)	1.39 (0.98–1.96)	1.33 (0.90–1.97)	0.110
Dairy	1.00 (reference)	0.90 (0.65–1.23)	0.98 (0.71–1.37)	1.22 (0.88–1.71)	0.203
Egg	1.00 (reference)	1.17 (0.87–1.57)	1.19 (0.88–1.61)	1.23 (0.88–1.71)	0.235
Fish	1.00 (reference)	0.77 (0.58–1.01)	0.63 (0.47–0.85)	0.80 (0.60–1.09)	0.089
Meat	1.00 (reference)	1.04 (0.77–1.41)	0.95 (0.69–1.32)	0.99 (0.69–1.41)	0.825
Plants	1.00 (reference)	**0.66 (0.49–0.88)**	**0.72 (0.53–0.96)**	**0.65 (0.47–0.91)**	**0.026**

^a^Values are hazard ratios (95% confidence intervals).

^b^Animal sources include contributions from meat, dairy, eggs, and fish.

Hazard ratios in bold represent statistically significant associations.

### 3.4 Subgroup analyses

In the analyses stratified by various demographic and lifestyle factors and medical histories, there were no materially change in the associations between total fat and SFAs from dairy intakes and the incidence of PCa (Supplementary Table S9). However, the inverse association of PUFAs from fish intake with PCa mortality was significant among older participants (≥65 years at DHQ completion) and those with alcohol consumption < median, for which all interactions were statistically significant (Supplementary Table S10). Although the association of PUFAs from fish intake with PCa mortality was stronger among participants with BMI ≥ 25, the *P* for interaction were not statistically different. None of the associations between fat quality index scores and MUFAs and SFAs from plant and PCa mortality changed significantly in the analyses stratified by age at baseline, alcohol consumption, BMI, smoking status, aspirin use, history of diabetes and trial group (Supplementary Tables S10, S11).

### 3.5 Sensitivity analyses

The initial association between total fat and SFAs from dairy and PCa incidence did not change substantially when excluding the first 2, 3, or 5 years of follow-up or extreme BMIs, further adjusting for Healthy Eating Index-2015 and physical activity or intakes of specific foods, or excluding participants who did not have complete covariate data (Supplementary Tables S12, S13). Similar to the full-scale analyses, the highest quartiles of MUFAs and SFAs from plants and PUFAs from fish intake were associated with higher mortality of PCa in sensitivity analysis strategies as compared to the lowest quartile (Supplementary Tables S14–S17).

## 4 Discussion

In this prospective cohort study, we found that long-term intake of total fat, trans fat, SFA, PUFA and MUFAs were not significantly associated with the PCa incidence and mortality. When considering various food sources of fat, we found that intake of dairy fat and SFAs was associated with an increased incidence of PCa in a linear dose-response manner. In contrast, intake of MUFAs and SFAs from plant source and PUFAs from fish were non-linearly associated with a lower mortality of PCa. Subgroup analyses further revealed a stronger reverse association for intake of PUFAs from fish in participants with age at DHQ completion ≥65 years or alcohol consumption below the median. These results were largely consistent in various sensitivity analyses, even after additional adjustment for physical activity and several markers of the nutritional quality of the diet. Therefore, our results support the hypothesis that intake of specific food sources of fat could be an important and modifiable factor for PCa incidence and mortality.

Previous research commonly believed that the associations between these macronutrients, including fat and health outcomes are linear within the range of consumption, and this association holds regardless of the level of intake of other macronutrients and total energy consumption (21, 22). However, our results demonstrated that the associations between dietary fat intake and PCa mortality were non-linear. These findings suggest the complex and diverse associations between fat intake and the incidence and mortality of PCa.

Based on our findings, dietary recommendations for primary prevention of PCa could consider emphasizing the intake of plant-based MUFAs and SFAs, as well as fish-based PUFAs. The exploration and development of nutritional supplements focused on these specific fatty acids, such as lauric acid and oleic acid, could provide targeted nutritional support. At the same time, it may be advisable to limit the intake of dairy products. However, these dietary recommendations should be tailored to individual needs, considering factors such as personal health status, dietary preferences, and cultural background.

## 5 Comparison with other studies and possible explanations

During the period of increasing high-fat intake, there has been a greater risk of overweight and adiposity in the adult American population (23–25). Consistent with this, the incidence and mortality of cancer is gradually increasing, and reducing fat intake are now considered critical but modifiable risk factors for various cancers (26–28). However, epidemiological studies examining this association remain limited. A previous large, international cohort consortium reported that high intakes of total and saturated fat were associated with an increased risk of lung cancer (HR quintile 5 vs. 1: 1.07 and 1.14, respectively; 95% CI: 1.00–1.15 and 1.07–1.22, respectively), especially among current smokers and for squamous cell and small cell carcinoma (29). Subsequently, a pooled analysis of 11 prospective cohort studies additionally provided new insights into the role of fat and oils in the incidence of bladder cancer, showing an inverse association between consumption of MUFAs and the development of bladder cancer among women (HR: 0.69, 95% CI: 0.53–0.91) and a direct association between higher intakes of dietary cholesterol and bladder cancer incidence among men (HR: 1.37, 95% CI: 1.16–1.61) (30). What is more, a Chinese prospective cohort reported that dietary intakes of total fat, SFAs, PUFAs, and probably MUFAs might increase liver cancer risks among men, implying the role of dietary nutrients on liver carcinogenesis (31).

Although the role of fat in PCa has been studied extensively, epidemiological studies remain inconsistent and inconclusive, and a consensus of the effects of fat on PCa risk has yet to be achieved. In this study, we observed that the quantity of fat was not associated with PCa risk. Consistent with these findings, a meta-analysis on 14 cohort studies, which included 37,349 cases and a total of 751,030 participants, reported no association between total fat, saturated fat, or unsaturated fat intake and PCa risk (12). However, a prospective NIH-AARP Diet and Health Study demonstrated that greater dietary intake of fat and fatty acids was not associated with the risk of non-advanced PCa, whereas intakes of saturated fat, ALA, and eicosapentaenoic acid (EPA) were associated with the risk of advanced or fatal PCa (32). Given these conflicting data, it is possible that the food sources of fat may play an important role in PCa development and progression. When condensing the food sources, we found that total fat and SFAs from dairy are associated with elevated risks of PCa incidence. Furthermore, some prospective cohort studies have also demonstrated that men with higher intake of dairy foods had a higher incidence of PCa compared with men having lower intake. The possible mechanism may be partially attributed to SFAs from dairy affecting the blood lipid profile and promoting tumorigenesis. The effects of dairy consumption on plasma Insulin-like growth factor (IGF), which predicts higher risks of PCa, provides another plausible mechanism (33).

Relatively few prospective studies have evaluated the associations of the quality and food sources of fat with PCa–specific mortality. We observed a stronger inverse association of intake of MUFAs and SFAs from plant and PUFAs from fish with the risk of PCa–specific mortality. The beneficial effects of MUFAs from plant may be partially attributed to a reduction in lipogenesis, an increase in β-oxidation, a decrease in intestinal cholesterol uptake, and enhancement of endothelial function (34, 35). Evidences from a Health Professionals Follow-up Study demonstrated that post-diagnostic vegetable fat was associated with lower risk of PCa mortality (HR [10% energy]: 0.71, 95% CI: 0.51–0.98) and all-cause mortality (HR [10% energy]: 0.74, 95% CI: 0.61–0.88) for men with non-metastatic PCa, implying that replacing carbohydrate and animal fat with vegetable fat may reduce risk of all-cause mortality (28). Increased fish consumption, particularly fatty fish, has also been related to reduced mortality, including all-cause mortality and cause-specific mortality and a total of 431,062 participants from the United Kingdom Biobank showed that consumption of fatty fish was inversely associated with risk for and overall mortality (36). Compared to other animal fats, fish fat has a high content of long-chain n-3 PUFAs, which may explain the beneficial role of fish fat in different health outcomes, including PCa (37, 38). Dietary sources of SFAs are animal products (butter, lard) and tropical plant oils (coconut, palm), and have been reported to be closely associated with the overall mortality, including PCa (39). However, we found no such associations and should exercise greater prudence in explaining this results. Regress of the potential benefits of plant-based diets, some studies reported that healthy plant-based diets can decrease the production of trimethylamine-N-oxide (the gut microbiota-produced metabolite of dietary phosphatidylcholine) (8), which has been found to be related to the development of PCa (40).

Consistent with our source-specific results, several biological pathways plausibly link dairy fat to higher PCa incidence and plant-derived fats and fish PUFAs to lower mortality. First, dairy fats are rich in SFAs, particularly myristic acid and palmitic acid, which has been shown to correlate positively with PCa risk in a dose-dependent manner (41). Myristic acid is metabolized into myristoyl-CoA, promoting Src kinase myristoylation and membrane localization. This activates phosphoinositide 3-kinase (PI3K)/Akt (protein kinase B) signaling, driving PCa growth and metastasis (42). Palmitic acid triggers epigenetic alterations in cancer cells, leading to the activation of Schwann cells, which in turn release extracellular matrix components that support metastatic initiation (43). Additionally, it stimulates nuclear factor kappa-light-chain-enhancer of activated B cells (NF-κB) signaling, contributing to the development of tumor-associated fibroblasts and promoting inflammatory responses (44). IGF-1 promotes cell proliferation and inhibits apoptosis. The intake of saturated fats increases serum IGF-1 levels, inhibits the binding of insulin-like growth factor-binding protein 3 (IGFBP-3) to IGF-1, and enhances the activity of free IGF-1, thereby promoting the progression of PCa (45). Activation of the IGF-1 signaling cascade stimulates mitogen-activated protein kinase (MAPK), which subsequently reduces the acetylation of heat shock protein 90, enhancing the binding of heat shock protein 90 to the androgen receptor. This process activates the androgen receptor, upregulating its associated proteins and genes, leading to an increase in androgen and testosterone levels. The elevated androgen levels further promote the development of PCa (46). Second, among the plant-derived SFAs, lauric acid and capric acid are more prevalent and exhibit strong antibacterial and anti-inflammatory effects. These effects are mediated through the inhibition of NF-κB activity and MAPK phosphorylation, which reduce the secretion of interleukin (IL)-6 and IL-8 (47). Lauric acid also suppresses the inflammatory response by inhibiting protein denaturation, proteinase activity, and the expression of oncogenic miRNAs (48). The Mediterranean diet is considered to have a slowing effect on PCa progression. This dietary pattern is characterized by olive oil's MUFA, oleic acid, as its main component, and it can reduce systemic inflammation levels (49). Third, fish rich in omega-3 PUFAs has the potential to inhibit PCa development by increasing the tumor suppressor protein PTEN and inhibiting PI3K activity. The effects of omega-3 fatty acids are controversial, with reports showing both positive and negative effects on IGF-1/PI3K/Akt (50, 51). However, Docosahexaenoic acid and EPA, have been found to inhibit the growth of PCa cells and may delay the progression of PCa to androgen-independent states by suppressing the mechanistic target of rapamycin signaling pathway and androgen receptor expression. This suggests that combining them with the current PCa treatment regimen androgen deprivation therapy, may enhance the treatment effectiveness.

## 6 Strengths and limitations

Strengths of our study pertain to its prospective design and large sample size, a substantial number of PCa cases and deaths along with a detailed assessment of dietary intake. We also explored associations with constituent sources of fat and fatty acids, including dairy, egg, fish, meat, and plants, each of which has distinctive relations with PCa incidence and morality. The possibility of confounding was dealt with through statistical adjustment for a wide range of covariates and through a series of sensitivity analyses. However, several limitations should be acknowledged. First, as with any observational study, we cannot exclude the possibility of residual confounding, including unmeasured behavioral factors and/or imprecision in the measure of included covariates, although the associations of dietary fat and fatty acids with PCa incidence and mortality were stable after adjustment for a wide range of potential confounding factors, including diabetes and BMI. Causality of the associations cannot be established from this observational cohort. Although randomized controlled trials have been considered ideal for elimination of confounding bias, they would not be conduct to investigate a deleterious effect due to ethical feasibility. Besides, randomized controlled trials do not capture consumption as it is in daily life, but this large observational cohort was particularly adapted to provide insights in this field. Second, dietary fat and fatty acids intake was assessed once at baseline in this study and may not reflect possible dietary modifications during follow-up, which may cause non-differential bias. Nonetheless, it has been proved that method only using baseline diet assessments typically result in a weaker association than that using the cumulative averages. Third, as over 90% participants in the PLCO cohort were non-Hispanic White, more than 60% had educational degree of some college or less; and almost half were aspirin users or ever smokers. This might limit the generalization of the findings. Finally, our study focuses on the specific effects of individual fat sources on PCa risk and mortality, rather than analyzing the broader dietary patterns. While this approach allows for a more detailed exploration of the relationship between specific fat types and PCa, it may limit the comprehensiveness of the overall analysis by not considering the potential interactions within the entire dietary patterns.

## 7 Conclusions and perspectives

In this large prospective cohort study, we demonstrated that a greater intake of MUFAs and SFAs from plant source and PUFAs from fish were associated with lower PCa mortality in a non-linear and linear manner, respectively. Besides, a high intake of dairy-based fat and SFAs is found to be associated with an elevated risk of PCa incidence in a linear manner. These findings highlight the importance of considering specific food sources of fat when evaluating the roles of dietary fat intake on PCa incidence and mortality, and provide detailed insights relevant to dietary guidelines that may support primary prevention of PCa. Our results need to be further validated in different populations and settings.

## Data Availability

The original contributions presented in the study are included in the article/Supplementary material, further inquiries can be directed to the corresponding authors.
