# Supplementary material for: Associations of specific food sources of dietary fat with prostate cancer incidence and mortality: results from a large prospective cohort
Source: Front Nutr. 2025 Nov 19;12:1630437. doi: 10.3389/fnut.2025.1630437 (PMC12672308; doi:10.3389/fnut.2025.1630437)
Supplement: Supplementary file 1 [file Table_1.docx]

**Associations of fat quality and sources with prostate cancer incidence and mortality: results from a large prospective cohort**

SUPPLEMENT 1.

eFigure 1. The flow chart of identifying individuals eligible for our study.

eTable 1. Comparison of sociodemographic characteristics between excluded and included populations

eTable 2. Distribution of variables with missing values before and after imputation

eTable 3. Baseline characteristics of the PLCO study population according to quarters of MUFAs intake

eTable 4. Baseline characteristics of the PLCO study population according to quarters of PUFAs intake

eTable 5. Baseline characteristics of the PLCO study population according to quarters of SFAs intake

eTable 6. Baseline characteristics of the PLCO study population according to quarters of TFAs intake

eTable 7. Association between dietary fat and specific fat type intake and the incidence of PCa.

eTable 8. Association between fat and specific fat type intake and the mortality of PCa.

eTable 9. Subgroup analyses on the association between fat and SFAs from dairy and the incidence of PCa

eTable 10. Subgroup analyses on the association between MUFAs and SFAs from plant PUFAs from fish and the mortality of PCa

eTable 11. Sensitivity analyses on the association between total fat from dairy and the incidence of PCa.

eTable 12. Sensitivity analyses on the association between SFAs from dairy and the incidence of PCa

eTable 13.Sensitivity analyses on the association between MUFAs from plants and the mortality of PCa.

eTable 14. Sensitivity analyses on the association between PUFAs from plants and the mortality of PCa.

eTable 15. Sensitivity analyses on the association between SFAs from plants and the mortality of PCa.





**eFigure 1.** The flow chart of identifying individuals eligible for our study.

| **eTable 1.** Comparison of sociodemographic characteristics between excluded and included populations ^1^ | | | |
| --- | --- | --- | --- |
| Sociodemographic characteristics | Excluded population | Included population | Standardized  difference |
| Number of participants | 27254 | 49424 |  |
| Age, years | 67.71 ± 5.84 | 65.78 ± 5.74 | 0.008 |
| Educational degree | | | |
| Some college or less | 15106 (62.1) | 28587(58.0) | 0.02 |
| College graduate | 4258 (17.5) | 9654 (19.6) |  |
| Postgraduate | 4967 (20.4) | 11070 (22.4) |  |
| Occupation | | | |
| Working | 10262(42.3) | 22205 (45.1) | 0.007 |
| Retired | 12187 (50.2) | 24462 (49.7) |  |
| Other | 1836 (7.6) | 2540 (6.0) |  |
| Smoking status | | | |
| Current | 3671(15.0) | 4987 (10.1) | 0.005 |
| Former | 12425 (50.9) | 25831(52.3) |  |
| Never | 8324 (34.1) | 18592 (37.6) |  |
| Number of cigarettes smoked per day | | | |
| 0 | 8324(34.2) | 185992 (37.7) | 0.013 |
| 1-10 | 2809 (11.5) | 5044 (10.2) |  |
| 11-20 | 5684 (23.3) | 11617 (23.5) |  |
| 21-30 | 3596 (14.8) | 7187 (14.6) |  |
| 31-40 | 2306 (9.5) | 4103 (8.3) |  |
| 41-60 | 1318 (5.4) | 2273 (4.6) |  |
| 61-80 | 256 (1.1) | 429 (0.9) |  |
| >80 | 78 (0.3) | 106 (0.2) |  |
| Body mass index ^2^ | 27.46± 4.33 | 27.51 ± 4.16 | 0.014 |
| Family history of prostate cancer | 1735 (7.2) | 3591 (7.3) | 0.002 |
| History of diabetes | 2726 (11.2) | 3949 (8.0) | 0.021 |
| Trial group | | | |
| Screening group | 12662 (46.5) | 25678 (52.0) | 0.006 |
| Control group | 14592 (53.5) | 23746 (48.0) |  |

^1^ Values are mean ± standard deviation or counts (percentage) as indicated. Standardized difference was calculated using the formula available in the paper by Austin PC. (DOI: 10.1080/00273171.2011.568786).

^2^ Weight (kg)/height (m)^2^.

| **eTable 2.** Distribution of variables with missing values before and after imputation ^1^ | | | |
| --- | --- | --- | --- |
| Covariates | Before imputation | After imputation | Number (%) with missing values |
| Educational level | | | |
| Some college or less | 28587 (57.97) | 28700(58.07) | 113 (0.23) |
| College graduate | 9654 (19.58) | 9654(19.53) |  |
| Postgraduate | 11070 (22.45) | 11070 (22.40) |  |
| Smoking status | | | |
| Current | 4987(10.09) | 4987(10.09) | 14 (0.03) |
| Former | 25831 (52.28) | 25845 (52.29) |  |
| Never | 18592 (37.63) | 18592 (37.62) |  |
| Number of cigarettes smoked per day | | | |
| 0 | 18592 (37.62) | 18665 (37.77) | 73(0.15) |
| 1-10 | 5044(10.22) | 5044(10.21) |  |
| 11-20 | 11617 (23.54) | 11617 (23.50) |  |
| 21-30 | 7187 (14.56) | 7187 (14.54) |  |
| 31-40 | 4103 (8.31) | 4103 (8.30) |  |
| 41-60 | 2273 (4.61) | 2273 (4.60) |  |
| 61-80 | 429 (0.87) | 429 (0.87) |  |
| >80 | 106 (0.21) | 106 (0.21) |  |
| Family history of prostate cancer | 3591 (7.33) | 3591 (7.27) | 434 (0.88) |
| History of diabetes | 3949(8.03) | 3949(7.99) | 274 (0.55) |
| Body mass index ^2^ | 27.50± 4.08 | 27.50 ± 4.05 | 726(1.47) |
| Physical activity level (min/week) ^3^ | 135.18 ±129.08 | 134.65 ±129.1 | 13602 (27.52) |

^1^ n = 49424. Values are mean (standard deviation) or counts (percentage) as indicated.

^2^ Weight (kg)/height (m)^2^.

^3^ Total time of moderate-to-vigorous physical activity per week

| **eTable 3.**  Baseline characteristics of the PLCO study population according to quarters of MUFAs intake (n = 49,424), *Values are mean ± SD or numbers (percentages) | | | | | | |
| --- | --- | --- | --- | --- | --- | --- |
| **Characteristics** | **Quarters of MUFAs intake** | | | | | ***P* for trend**^1^ |
|  | **All participants** | **Q1** (n = 12,356) | **Q2** (n = 12,356) | **Q3** (n = 12,356) | **Q4** (n = 12,356) |  |
| Age, years | 65.8 (5.7) | 66.2 (5.8) | 66.3 (5.8) | 65.7 (5.7) | 65.0 (5.5) | <0.001 |
| Body mass index (kg/m^2^) | 27.5 (4.1) | 26.9 (3.8) | 27.3 (3.9 | 27.7 (4.1) | 28.2 (4.4) | <0.001 |
| Physical activity (min/week)^2^ | 134.7 (129.1) | 148.8 (135.4) | 135.2 (127.0) | 126.3 (124.9) | 128.2 (127.8) | <0.001 |
| Family history of prostate cancer, n (%) | 3,591 (7.3%) | 876 (7.1%) | 902 (7.3%) | 883 (7.1%) | 930 (7.5%) | 0.3 |
| History of diabetes, n (%) | 3,949 (8.0%) | 718 (5.8%) | 976 (7.9%) | 1,016 (8.2%) | 1,239 (10.0%) | <0.001 |
| Aspirin user, n (%) | 25,606 (52.2%) | 6,697 (54.6%) | 6,380 (52.0%) | 6,210 (50.6%) | 6,319 (51.5%) | <0.001 |
| **Racial/ethnic group, n (%)** |  |  |  |  |  | <0.001 |
| Non-Hispanic White | 44,846 (90.7%) | 11,062 (89.5%) | 11,153 (90.3%) | 11,305 (91.5%) | 11,326 (91.7%) |  |
| Non-Hispanic Black | 1,341 (2.7%) | 333 (2.7%) | 327 (2.6%) | 348 (2.8%) | 333 (2.7%) |  |
| Hispanic | 864 (1.7%) | 156 (1.3%) | 226 (1.8%) | 215 (1.7%) | 267 (2.2%) |  |
| Other race/ethnicity3 | 2,373 (4.8%) | 805 (6.5%) | 650 (5.3%) | 488 (3.9%) | 430 (3.5%) |  |
| **Educational degree, n (%)** |  |  |  |  |  | <0.001 |
| Postgraduate | 11,070 (22.4%) | 3,355 (27.2%) | 2,913 (23.6%) | 2,526 (20.4%) | 2,276 (18.4%) |  |
| College graduate | 9,654 (19.5%) | 2,579 (20.9%) | 2,513 (20.3%) | 2,353 (19.0%) | 2,209 (17.9%) |  |
| College below | 28,700 (58.1%) | 6,422 (52.0%) | 6,930 (56.1%) | 7,477 (60.5%) | 7,871 (63.7%) |  |
| **Smoking status, n (%)** |  |  |  |  |  | <0.001 |
| Never | 18,592 (37.6%) | 4,873 (39.4%) | 4,941 (40.0%) | 4,669 (387.%) | 4,109 (33.3%) |  |
| Current | 4,987 (10.1%) | 950 (7.7%) | 982 (7.9%) | 1,299 (10.5%) | 1,756 (14.2%) |  |
| Former | 25,845 (52.3%) | 6,533 (52.9%) | 6,433 (52.1%) | 6,388 (51.7%) | 6,491 (52.5%) |  |
| Alcohol intake, g/d | 14.3 (33.6) | 30.0 (59.5) | 10.7 (16.9) | 8.4 (13.5) | 8.0 (13.2) | <0.001 |
| Energy intake from diet, kcal/day | 1,994.5 (814.5) | 2,130.3 (832.7) | 1,726.6 (676.2) | 1,790.9 (719.3) | 2,330.1 (861.0) | <0.001 |
| Healthy Eating Index-2015 | 66.5 (9.7) | 69.3 (9.0) | 66.8 (8.7) | 63.0 (9.0) | 59.4 (9.5) | <0.001 |
| **Food consumption** |  |  |  |  |  |  |
| Whole grain (g/day) | 66.1 (66.0) | 89.0 (83.5) | 68.2 (62.5) | 55.5 (53.6) | 51.7 (53.4) | <0.001 |
| Vegetable (g/day) | 290.0 (193.1) | 341.5 (235.3) | 265.5 (170.5) | 255.6 (166.6) | 297.5 (180.2) | <0.001 |
| Fruit (g/day) | 265.6 (223.1) | 387.7 (307.5) | 265.0 (176.3) | 212.9 (158.3) | 196.9 (161.4) | <0.001 |
| Red Meats, processed (g/day) | 17.1 (18.6) | 11.4 (13.4) | 12.8 (12.7) | 16.5 (15.2) | 27.6 (25.5) | <0.001 |
| Red Meats, not processed (g/day) | 62.2 (50.8) | 46.1 (38.4) | 49.8 (37.0) | 60.9 (42.5) | 91.9 (66.2) | <0.001 |
| White Meats (g/day) | 52.0 (49.3) | 56.3 (53.0) | 45.6 (43.0) | 47.0 (43.8) | 58.9 (55.2) | <0.001 |
| **Nutrient intake** |  |  |  |  |  |  |
| SFAs (g/day) | 23.4 (13.1) | 17.9 (9.6) | 18.9 (9.8) | 22.6 (10.6) | 34.1 (15.1) | <0.001 |
| Fat (g/day) | 72.5 (37.4) | 56.2 (26.7) | 58.2 (26.9) | 69.3 (28.9) | 106.2 (41.4) | <0.001 |
| PUFAs (g/day) | 27.7 (14.7) | 12.8 (6.3) | 12.8 (6.1) | 14.9 (6.7) | 22.5 (9.5) | <0.001 |
| Carbohydrate (g/day) | 245.7 (98.9) | 291.6 (105.1) | 226.2 (84.1) | 216.8 (89.2) | 248.1 (98.7) | <0.001 |
| TFAs (g/day) | 4.7 (2.7) | 3.7 (2.0) | 3.8 (2.0) | 4.6 (2.2) | 6.6 (3.2) | <0.001 |
| Protein (g/day) | 75.9 (33.6) | 75.6 (30.7) | 65.9 (28.3) | 69.7 (30.2) | 92.4 (38.1) | <0.001 |
| Cholesterol (mg/day) | 247.8 (151.8) | 193.0 (113.0) | 201.0 (112.7) | 240.3 (123.3) | 356.8 (185.3) | <0.001 |
| Dietary fiber (g/day) | 19.2 (9.1) | 23.2 (10.3) | 17.7 (7.7) | 16.6 (7.8) | 19.5 (8.8) | <0.001 |
| Sodium (mg/day) | 3,121.0 (1340.6) | 3,089.7 (1,250.1) | 2,737.4 (1,146.3) | 2,889.4 (1,222.5) | 3,767.5 (1,482.6) | <0.001 |
| **Body mass index, n (%)** |  |  |  |  |  | <0.001 |
| Underweight (<18.5 kg/m^2^) | 131(0.3%) | 34 (0.3%) | 26 (0.2%) | 39 (0.3%) | 32 (0.3%) |  |
| Normal (18.5-24.9 kg/m^2^) | 12,733(25.8%) | 3,860 (31.2%) | 3,345 (27.1%) | 2,920 (23.6%) | 2,608 (21.1%) |  |
| Overweight (25-29.9 kg/m^2^) | 25,614(51.8%) | 6,364 (51.5%) | 6,526 (52.8%) | 6,506 (52.7%) | 6,218 (50.3%) |  |
| Obese(>30 kg/m^2^) | 10,946(22.1%) | 2,098 (17.0%) | 2,459 (19.9%) | 2,891 (23.4%) | 3,498 (28.3%) |  |
| ^1^ *P* value for comparison between quarters of total fat intake, by Kruskal-Wallis rank sum test or pearson's Chi-squared test.  ^2^ Total time of moderate-to-vigorous physical activity per week.  ^3^ Other race/ethnicity = Asian, Pacific Islander or American Indian. | | | | | | |

| **eTable 4.**  Baseline characteristics of the PLCO study population according to quarters of PUFAs intake (n = 49,424), *Values are mean ± SD or numbers (percentages) | | | | | | |
| --- | --- | --- | --- | --- | --- | --- |
| **Characteristics** | **Quintile of PUFAs intake** | | | | | ***P* for trend**^2^ |
|  | **All participants** | **Q1** (n = 12,356) | **Q2** (n = 12,356) | **Q3** (n = 12,356) | **Q4** (n = 12,356) |  |
| Age, years | 65.8 (5.7) | 65.9 (5.7) | 66.1 (5.8) | 65.8 (5.7) | 65.3 (5.6) | <0.001 |
| Body mass index (kg/m^2^) | 27.5 (4.1) | 27.2 (3.9) | 27.5 (4.0) | 27.6 (4.0) | 27.8 (4.2) | <0.001 |
| Physical activity (min/week)^4^ | 134.7 (129.1) | 139.9 (133.9) | 132.4 (125.4) | 130.1 (125.6) | 136.3 (131.2) | <0.001 |
| Family history of prostate cancer, n (%) | 3,591 (7.3%) | 901 (7.3%) | 880 (7.1%) | 920 (7.4%) | 890 (7.2%) | >0.9 |
| History of diabetes, n (%) | 3,949 (8.0%) | 704 (5.7%) | 933 (7.6%) | 1,096 (8.9%) | 1,216 (9.8%) | <0.001 |
| Aspirin user, n (%) | 25,606 (52.2%) | 6,506 (52.9%) | 6,480 (52.8%) | 6,325 (51.5%) | 6,295 (51.3%) | 0.016 |
| **Racial/ethnic group, n (%)** |  |  |  |  |  | <0.001 |
| Non-Hispanic White | 44,846 (90.7%) | 11,409 (92.3%) | 11,321 (91.6%) | 11,213 (90.7%) | 10,903 (88.2%) |  |
| Non-Hispanic Black | 1,341 (2.7%) | 268 (2.2%) | 313 (2.5%) | 333 (2.7%) | 427 (3.5%) |  |
| Hispanic | 864 (1.7%) | 216 (1.7%) | 237 (1.9%) | 215 (1.7%) | 196 (1.6%) |  |
| Other race/ethnicity | 2,373 (4.8%) | 463 (3.7%) | 485 (3.9%) | 595 (4.8%) | 830 (6.7%) |  |
| **Educational degree, n (%)** |  |  |  |  |  | 0.10 |
| Postgraduate | 11,070 (22.4%) | 2,786 (22.5%) | 2,804 (22.7%) | 2,770 (22.4%) | 2,710 (21.9%) |  |
| College graduate | 9,654 (19.5%) | 2,319 (18.8%) | 2,435 (19.7%) | 2,396 (19.4%) | 2,504 (20.3%) |  |
| College below | 28,700 (58.1%) | 7,251 (58.7%) | 7,117 (57.6%) | 7,190 (58.2%) | 7,142 (57.8%) |  |
| **Smoking status, n (%)** |  |  |  |  |  | <0.001 |
| Never | 18,592 (37.6%) | 4,687 (37.9%) | 4,802 (38.9%) | 4,665 (37.8%) | 4,438 (35.9%) |  |
| Current | 4,987 (10.1%) | 1,347 (10.9%) | 1,129 (9.1%) | 1,219 (9.9%) | 1,292 (10.5%) |  |
| Former | 25,845 (52.3%) | 6,322 (51.2%) | 6,425 (52.0%) | 6,472 (52.4%) | 6,626 (53.6%) |  |
| Alcohol intake, g/d | 14.3 (33.6) | 29.1 (59.0) | 10.2 (16.9) | 8.8 (14.7) | 9.1 (15.3) | <0.001 |
| Energy intake from diet, kcal/day | 1,994.5 (814.5) | 2,199.0 (867.7 | 1,726.0 (692.0) | 1,805.7 (712.0) | 2,247.2 (837.4) | <0.001 |
| Healthy Eating Index-2015 | 66.5 (9.7) | 64.3 (10.2) | 65.0 (9.8) | 64.8 (9.8) | 64.5 (9.5) | <0.001 |
| **Food consumption** |  |  |  |  |  |  |
| Whole grain (g/day) | 66.1 (66.0) | 76.7 (76.2) | 64.1 (61.2) | 61.2 (61.5) | 62.5 (62.9) | <0.001 |
| Vegetable (g/day) | 290.0 (193.1) | 299.8 (210.4) | 258.5 (172.8) | 269.9 (173.7) | 331.9 (203.9) | <0.001 |
| Fruit (g/day) | 265.6 (223.1) | 338.4 (296.5) | 254.4 (187.8) | 233.1 (178.8) | 236.8 (191.6) | <0.001 |
| Red Meats, processed (g/day) | 17.1 (18.6) | 16.1 (18.6) | 14.6 (15.9) | 16.4 (17.0) | 21.2 (21.8) | <0.001 |
| Red Meats, not processed (g/day) | 62.2 (50.8) | 62.3 (54.9) | 55.3 (44.5) | 58.8 (45.0) | 72.4 (56.0) | <0.001 |
| White Meats (g/day) | 52.0 (49.3) | 47.7 (44.8) | 45.4 (41.9) | 50.3 (45.7) | 64.5 (60.7) | <0.001 |
| **Nutrient intake** |  |  |  |  |  |  |
| SAFs (g/day) | 23.4 (13.1) | 22.8 (13.8) | 19.8 (11.2) | 21.9 (11.3) | 29.2 (14.1) | <0.001 |
| Fat (g/day) | 72.5 (37.4) | 64.2 (34.0) | 59.3 (30.1) | 68.6 (30.9) | 97.9 (41.1) | <0.001 |
| MUFAs (g/day) | 27.7 (14.7) | 24.3 (13.1) | 22.7 (11.8) | 26.3 (12.2) | 37.6 (16.5) | <0.001 |
| Carbohydrate (g/day) | 245.7 (98.9) | 287.1 (106.7) | 222.7 (85.5) | 221.2 (88.5) | 251.6 (98.7) | <0.001 |
| TFAs (g/day) | 4.7 (2.7) | 4.3 (2.4) | 4.0 (2.3) | 4.5 (2.4) | 5.9 (3.1) | <0.001 |
| Protein (g/day) | 75.9 (33.6) | 79.4 (33.7) | 67.3 (29.7) | 70.4 (30.5) | 86.5 (36.6) | <0.001 |
| Cholesterol (mg/day) | 247.8 (151.8) | 236.4 (147.0) | 213.5 (129.4) | 238.6 (136.9) | 302.6 (175.1) | <0.001 |
| Dietary fiber (g/day) | 19.2 (9.1) | 21.1 (9.9) | 17.2 (8.0) | 17.5 (8.1) | 21.1 (9.4) | <0.001 |
| Sodium (mg/day) | 3121.0 (1340.6) | 3,130.9 (1,308.1) | 2,753.3 (1,192.9) | 2,937.1 (1,228.5) | 3,662.7 (1,443.4) | <0.001 |
| **Body mass index, n (%)** |  |  |  |  |  | <0.001 |
| Underweight (<18.5 kg/m^2^) | 131(0.3%) | 37 (0.3%) | 29 (0.2%) | 34 (0.3%) | 31 (0.3%) |  |
| Normal (18.5-24.9 kg/m^2^) | 12,733(25.8%) | 3,552 (28.7%) | 3,184 (25.8%) | 3,037 (24.6%) | 2,960 (24.0%) |  |
| Overweight (25-29.9 kg/m^2^) | 25,614(51.8%) | 6,366 (51.5%) | 6,544 (53.0%) | 6,372 (51.6%) | 6,332 (51.2%) |  |
| Obese(>30 kg/m^2^) | 10,946(22.1%) | 2,401 (19.4%) | 2,599 (21.0%) | 2,913 (23.6%) | 3,033 (24.5%) |  |
| ^1^Mean (SD; n (%) | | | | | | |
| ^2^Kruskal-Wallis rank sum test; Pearson's Chi-squared test | | | | | | |

| **eTable 5.** Baseline characteristics of the PLCO study population according to quarters of SFAs intake (n = 49,424), *Values are mean ± SD or numbers (percentages) | | | | | | |
| --- | --- | --- | --- | --- | --- | --- |
| **Characteristics** | **Quarters of SFAs intake** | | | | | ***P* for trend**^2^ |
|  | **All participants** | **Q1** (n = 12,356) | **Q2** (n = 12,358) | **Q3** (n = 12,354) | **Q4** (n = 12,356) |  |
| Age, years | 65.8 (5.7) | 65.6 (5.7) | 66.0 (5.8) | 66.0 (5.8) | 65.5 (5.7) | <0.001 |
| Body mass index (kg/m^2^) | 27.5 (4.1) | 26.9 (3.9) | 27.3 (3.9) | 27.8 (4.0) | 28.1 (4.3) | <0.001 |
| Physical activity (min/week)^4^ | 134.7 (129.1) | 151.8 (137.0) | 138.3 (128.1) | 127.2 (124.0) | 121.3 (124.8) | <0.001 |
| Family history of prostate cancer, n (%) | 3,591 (7.3%) | 909 (7.4%) | 919 (7.4%) | 913 (7.4%) | 850 (6.9%) | 0.002 |
| History of diabetes, n (%) | 3,949 (8.0%) | 776 (6.3%) | 909 (7.4%) | 1,100 (8.9%) | 1,164 (9.4%) | <0.001 |
| Aspirin user, n (%) | 25,606 (52.2%) | 6,550 (53.0%) | 6,447 (52.1%) | 6,320 (51.1%) | 6,289 (50.9%) | 0.003 |
| **Racial/ethnic group, n (%)** |  |  |  |  |  | <0.001 |
| Non-Hispanic White | 44,846 (90.7%) | 10,745 (87.0%) | 11,154 (90.2%) | 11,386 (92.1%) | 11,561 (93.6%) |  |
| Non-Hispanic Black | 1,341 (2.7%) | 305 (2.5%) | 255 (2.1%) | 361 (2.9%) | 420 (3.4%) |  |
| Hispanic | 864 (1.7%) | 202 (1.6%) | 206 (1.7%) | 242 (2.0%) | 214 (1.7%) |  |
| Other race/ethnicity | 2,373 (4.8%) | 1,104 (8.9%) | 743 (6.0%) | 365 (3.0%) | 161 (1.3%) |  |
| **Educational degree, n (%)** |  |  |  |  |  | <0.001 |
| Postgraduate | 11,070 (22.4%) | 3,602 (29.1%) | 3,143 (25.4%) | 2,487 (20.1%) | 1,838 (14.9%) |  |
| College graduate | 9,654 (19.5%) | 2,770 (22.4%) | 2,637 (21.3%) | 2,300 (18.6%) | 1,947 (15.7%) |  |
| College below | 28,700 (58.1%) | 5,984 (48.5%) | 6,578 (53.3%) | 7,567 (61.3%) | 8,571 (69.4%) |  |
| **Smoking status, n (%)** |  |  |  |  |  | <0.001 |
| Never | 18,592 (37.6%) | 4,770 (39%) | 4,901 (40%) | 4,622 (37%) | 4,299 (35%) |  |
| Current | 4,987 (10.1%) | 1,097 (8.9%) | 1,016 (8.2%) | 1,248 (10%) | 1,626 (13%) |  |
| Former | 25,845 (52.3%) | 6,489 (53%) | 6,441 (52%) | 6,484 (52%) | 6,431 (52%) |  |
| Alcohol intake, g/d | 14.3 (33.6) | 31.7 (59.4) | 11.4 (16.7) | 7.7 (12.2) | 6.4 (11.7) | <0.001 |
| Energy intake from diet, kcal/day | 1,994.5 (814.5) | 2,401.7 (843.7) | 1,833.4 (664.3) | 1,695.1 (709.6) | 2,047.6 (844.2) | <0.001 |
| Healthy Eating Index-2015 | 66.5 (9.7) | 69.2 (9.4) | 66.7 (8.9) | 63.5 (8.8) | 59.0 (9.1) | <0.001 |
| **Food consumption** |  |  |  |  |  |  |
| Whole grain (g/day) | 66.1 (66.0) | 87.5 (85.7) | 67.6 (60.4) | 56.0 (52.9) | 53.4 (54.2) | <0.001 |
| Vegetable (g/day) | 290.0 (193.1) | 369.5 (243.0 | 278.5 (170.3) | 244.6 (155.7) | 267.5 (166.8) | <0.001 |
| Fruit (g/day) | 265.6 (223.1) | 388.7 (310.7 | 271.3 (182.4) | 214.0 (150.3) | 188.5 (151.5) | 0.038 |
| Red Meats, processed (g/day) | 17.1 (18.6) | 15.9 (19.1) | 15.3 (17.1) | 15.9 (16.4) | 21.3 (20.9) | <0.001 |
| Red Meats, not processed (g/day) | 62.2 (50.8) | 59.3 (51.4) | 56.2 (45.1) | 58.3 (46.3) | 74.9 (57.2) | <0.001 |
| Whole grain (g/day) | 52.0 (49.3) | 61.9 (56.4) | 49.8 (45.1) | 44.2 (41.5) | 52.0 (51.5) | <0.001 |
| **Nutrient intake** |  |  |  |  |  |  |
| Fat (g/day) | 72.5 (37.4) | 75.2 (38.1) | 64.8 (32.7 | 64.3 (33.4) | 85.7 (40.7) | <0.001 |
| PUFAs (g/day) | 15.7 (8.3) | 17.2 (9.1 | 14.1 (7.1) | 13.6 (7.1) | 18.0 (8.8) | <0.001 |
| MUFAs(g/day) | 27.7 (14.7) | 28.5 (15.1) | 24.5 (12.8) | 24.5 (13.0) | 33.3 (16.0) | <0.001 |
| Carbohydrate (g/day) | 245.7 (98.9) | 303.6 (104.4) | 232.0 (80.5 | 208.4 (83.4) | 238.7 (99.2) | <0.001 |
| TFAs (g/day) | 4.7 (2.7) | 2.2 (0.9) | 3.7 (1.1) | 5.0 (1.5) | 7.8 (2.8) | <0.001 |
| Protein (g/day) | 75.9 (33.6) | 87.2 (34.6) | 71.4 (29.6) | 66.5 (30.4) | 78.5 (35.6) | <0.001 |
| Cholesterol (mg/day) | 247.8 (151.8) | 242.1 (148.6) | 223.1 (135.7) | 226.8 (135.5) | 299.1 (172.0) | <0.001 |
| Dietary fiber (g/day) | 19.2 (9.1) | 24.9 (10.4) | 18.4 (7.3) | 16.0 (7.2) | 17.7 (8.2) | <0.001 |
| Sodium (mg/day) | 3121.0 (1340.6) | 3,469.8 (1,352.2) | 2,917.6 (1,191.4) | 2,751.1 (1,220.1) | 3,345.5 (1,450.1) | <0.001 |
| **Body mass index, n (%)** |  |  |  |  |  | <0.001 |
| Underweight (<18.5 kg/m^2^) | 131(0.3%) | 38 (0.3%) | 33 (0.3%) | 34 (0.3%) | 26 (0.2%) |  |
| Normal (18.5-24.9 kg/m^2^) | 12,733(25.8%) | 3,878 (31.4%) | 3,383 (27.4%) | 2,792 (22.6%) | 2,680 (21.7%) |  |
| Overweight (25-29.9 kg/m^2^) | 25,614(51.8%) | 6,276 (50.8%) | 6,429 (52.0%) | 6,614 (53.5%) | 6,295 (50.9%) |  |
| Obese(>30 kg/m^2^) | 10,946(22.1%) | 2,164 (17.5%) | 2,513 (20.3%) | 2,914 (23.6%) | 3,355 (27.2%) |  |
| ^1^Mean (SD; n (%) | | | | | | |
| ^2^Kruskal-Wallis rank sum test; Pearson's Chi-squared test | | | | | | |

| **eTable 6.** Baseline characteristics of the PLCO study population according to quarters of TFAs intake (n = 49424), *Values are mean ± SD or numbers (percentages) | | | | | | |
| --- | --- | --- | --- | --- | --- | --- |
| **Characteristics** | **Quintile of TFAs intake** | | | | | ***P* for trend**^1^ |
|  | **All participants** | **Q1** (n = 12,356) | **Q2** (n = 12,358) | **Q3** (n = 12,354) | **Q4** (n = 12,356) |  |
| Age, years | 65.8 (5.7) | 65.6 (5.7) | 66.0 (5.8) | 66.0 (5.8) | 65.5 (5.7) | <0.001 |
| Body mass index (kg/m^2^) | 27.5 (4.1) | 26.9 (3.8) | 27.3 (3.9) | 27.8 (4.0) | 28.1 (4.3) | <0.001 |
| Physical activity (min/week)^2^ | 134.7 (129.1) | 151.8 (137.0) | 138.3 (128.1) | 127.2 (124.0) | 121.3 (124.8) | <0.001 |
| Family history of prostate cancer, n (%) | 3,591 (7.3%) | 909 (7.4%) | 919 (7.4%) | 913 (7.4%) | 850 (6.9%) | 0.002 |
| History of diabetes, n (%) | 3,949 (8.0%) | 776 (6.3%) | 909 (7.4%) | 1,100 (8.9%) | 1,164 (9.4%) |  |
| Aspirin user, n (%) | 25,606 (52.2%) | 6,550 (53.3%) | 6,447 (52.6%) | 6,320 (51.5%) | 6,289 (51.2%) |  |
| **Racial/ethnic group, n (%)** |  |  |  |  |  | <0.001 |
| Non-Hispanic White | 44,846 (90.7%) | 10,745 (87.0%) | 11,154 (90.3%) | 11,386 (92.2%) | 11,561 (93.6%) |  |
| Non-Hispanic Black | 1,341 (2.7%) | 305 (2.5%) | 255 (2.1%) | 361 (2.9%) | 420 (3.4%) |  |
| Hispanic | 864 (1.7%) | 202 (1.6%) | 206 (1.7%) | 242 (2.0%) | 214 (1.7%) |  |
| Other race/ethnicity3 | 2,373 (4.8%) | 1,104 (8.9%) | 743 (6.0%) | 365 (3.0%) | 161 (1.3%) |  |
| **Educational degree, n (%)** |  |  |  |  |  | <0.001 |
| Postgraduate | 11,070 (22.4%) | 3,602 (29.2%) | 3,143 (25.4%) | 2,487 (20.1%) | 1,838 (14.9%) |  |
| College graduate | 9,654 (19.5%) | 2,770 (22.4%) | 2,637 (21.3%) | 2,300 (18.6%) | 1,947 (15.8%) |  |
| College below | 28,700 (58.1%) | 5,984 (48,4%) | 6,578 (53.2%) | 7,567 (61.3%) | 8,571 (69.4%) |  |
| **Smoking status, n (%)** |  |  |  |  |  | <0.001 |
| Never | 18,592 (37.6%) | 4,770 (38.6%) | 4,901 (39.7%) | 4,622 (37.4%) | 4,299 (34.8%) |  |
| Current | 4,987 (10.1%) | 1,097 (8.9%) | 1,016 (8.2%) | 1,248 (10.1%) | 1,626 (13.2%) |  |
| Former | 25,845 (52.3%) | 6,489 (52.5%) | 6,441 (52.1%) | 6,484 (52.5%) | 6,431 (52.0%) |  |
| Alcohol intake, g/d | 14.3 (33.6) | 31.7 (59.4) | 11.4 (16.7) | 7.7 (12.2) | 6.4 (11.7) | <0.001 |
| Energy intake from diet, kcal/day | 1,994.5 (814.5) | 2,401.7 (843.7) | 1,833.4 (664.3) | 1,695.1 (709.6) | 2,047.6 (844.2) | <0.001 |
| Healthy Eating Index-2015 | 66.5 (9.7) | 69.2 (9.4) | 66.7 (8.9) | 63.5 (8.8) | 59.0 (9.1) | <0.001 |
| **Food consumption** |  |  |  |  |  |  |
| Whole grain (g/day) | 66.1 (66.0) | 87.5 (85.7) | 67.6 (60.4) | 56.0 (52.9) | 53.4 (54.2) | <0.001 |
| Vegetable (g/day) | 290.0 (193.1) | 369.5 (243.0) | 278.5 (170.3) | 244.6 (155.7) | 267.5 (166.8) | <0.001 |
| Fruit (g/day) | 265.6 (223.1) | 388.7 (310.7) | 271.3 (182.4) | 214.0 (150.3) | 188.5 (151.5) | <0.001 |
| Red Meats, processed (g/day) | 17.1 (18.6) | 15.9 (19.1) | 15.3 (17.1) | 15.9 (16.4) | 21.3 (20.9) | <0.001 |
| Red Meats, not processed (g/day) | 62.2 (50.8) | 59.3 (51.4) | 56.2 (45.1) | 58.3 (46.3) | 74.9 (57.2) | <0.001 |
| White Meats (g/day) | 52.0 (49.3) | 61.9 (56.4) | 49.8 (45.1) | 44.2 (41.5) | 52.0 (51.5) | <0.001 |
| **Nutrient intake** |  |  |  |  |  |  |
| Fat (g/day) | 72.5 (37.4) | 75.2 (38.1) | 64.8 (32.7) | 64.3 (33.4) | 85.7 (40.7) | <0.001 |
| PUFAs (g/day) | 15.7 (8.3) | 17.2 (9.1) | 14.1 (7.1) | 13.6 (7.1) | 18.0 (8.8) | <0.001 |
| MUFAs (g/day) | 27.7 (14.7) | 28.5 (15.1) | 24.5 (12.8) | 24.5 (13.0) | 33.3 (16.0) | <0.001 |
| SFAs (g/day) | 23.4 (13.1) | 23.6 (13.3) | 21.0 (11.8) | 21.1 (11.9) | 27.9 (14.2) | <0.001 |
| Carbohydrate (g/day) | 75.9 (33.6) | 303.6 (104.4) | 232.0 (80.5) | 208.4 (83.4) | 238.7 (99.2) | <0.001 |
| Protein (g/day) | 247.8 (151.8) | 87.2 (34.6) | 71.4 (29.6) | 66.5 (30.4) | 78.5 (35.6) | <0.001 |
| Dietary fiber (g/day) | 19.2 (9.1) | 24.9 (10.4) | 18.4 (7.3) | 16.0 (7.2) | 17.7 (8.2) | <0.001 |
| Sodium (mg/day) | 3,121.0 (1340.6) | 3,469.8 (1,352.2) | 2,917.6 (1,191.4) | 2,751.1 (1,220.1) | 3,345.5 (1,450.1) | <0.001 |
| **Body mass index, n (%)** | 49,424 |  |  |  |  | <0.001 |
| Underweight (<18.5 kg/m^2^) |  | 38 (0.3%) | 33 (0.3%) | 34 (0.3%) | 26 (0.2%) |  |
| Normal (18.5-24.9 kg/m^2^) |  | 3,878 (31.4%) | 3,383 (27.4%) | 2,792 (22.6%) | 2,680 (21.7%) |  |
| Overweight (25-29.9 kg/m^2^) |  | 6,276 (50.8%) | 6,429 (52.0%) | 6,614 (53.5%) | 6,295 (50.9%) |  |
| Obese(>30 kg/m^2^) |  | 2,164 (17.5%) | 2,513 (20.3%) | 2,914 (23.6%) | 3,355 (27.2%) |  |
| ^1^ *P* value for comparison between quarters of total fat intake, by Kruskal-Wallis rank sum test or pearson's Chi-squared test.  ^2^ Total time of moderate-to-vigorous physical activity per week.  ^3^ Other race/ethnicity = Asian, Pacific Islander or American Indian. | | | | | | |

| **eTable 7.** Association between dietary fat and specific fat type intake and the incidence of PCa^a^. | | | | | |
| --- | --- | --- | --- | --- | --- |
| **Categories and model** | **Quartile 1** | **Quartile 2** | **Quartile 3** | **Quartile 4** | ***P-*trend** |
| **Total fat, g. day^-1^.kcal^-1^** | <63.36 | 63.36-72.75 | 72.75-82.25 | >82.25 |  |
| Cases, n | 1070 | 1107 | 1081 | 1050 |  |
| Unadjusted | 1.00 (reference) | 1.05 (0.96-1.14) | 1.03 (0.95-1.12) | 1.01 (0.93-1.10) | 0.925 |
| Model 1 | 1.00 (reference) | 1.04 (0.95-1.13) | 1.03 (0.95-1.12) | 1.02 (0.93-1.11) | 0.756 |
| Model 2 | 1.00 (reference) | 1.05 (0.97-1.15) | 1.07 (0.98-1.17) | 1.10 (1.00-1.20) | 0.058 |
| Model 3 | 1.00 (reference) | 1.05 (0.95-1.16) | 1.06 (0.94-1.20) | 1.09 (0.93-1.28) | 0.312 |
| **MUFAs, g. day^-1^.kcal^-1^** | <23.86 | 23.86-27.70 | 27.70-31.64 | >31.64 |  |
| Cases, n | 1098 | 1095 | 1084 | 1031 |  |
| Unadjusted | 1.00 (reference) | 1.01 (0.93-1.09) | 1.01 (0.92-1.09) | 0.96 (0.88-1.05) | 0.408 |
| Model 1^b^ | 1.00 (reference) | 1.00 (0.92-1.09) | 1.00 (0.92-1.09) | 0.97 (0.89-1.06) | 0.609 |
| Model 2^c^ | 1.00 (reference) | 1.01 (0.92-1.10) | 1.04 (0.95-1.13) | 1.04 (0.95-1.14) | 0.349 |
| Model 3^d^ | 1.00 (reference) | 0.97 (0.88-1.07) | 0.98 (0.87-1.10) | 0.95 (0.82-1.10) | 0.525 |
| **PUFAs, g. day^-1^.kcal^-1^** | <13.04 | 13.04-15.27 | 15.27-18.00 | >18.00 |  |
| Cases, n | 1077 | 1082 | 1097 | 1052 |  |
| Unadjusted | 1.00 (reference) | 1.00 (0.92-1.09) | 1.03 (0.94-1.12) | 0.98 (0.90-1.07) | 0.792 |
| Model 1^b^ | 1.00 (reference) | 1.00 (0.92-1.09) | 1.03 (0.95-1.12) | 1.00 (0.92-1.09) | 0.852 |
| Model 2^c^ | 1.00 (reference) | 1.01 (0.92-1.10) | 1.05 (0.96-1.15) | 1.03 (0.94-1.12) | 0.392 |
| Model 3^d^ | 1.00 (reference) | 1.01 (0.92-1.10) | 1.05 (0.95-1.15) | 1.03 (0.92-1.15) | 0.507 |
| **SFAs, g. day^-1^.kcal^-1^** | <39.84 | 39.84-46.28 | 46.28-55.06 | >55.06 |  |
| Cases, n | 1056 | 1119 | 1047 | 1086 |  |
| Unadjusted | 1.00 (reference) | 1.08 (0.99-1.17) | 1.02 (0.93-1.11) | 1.07 (0.98-1.16) | 0.323 |
| Model 1^b^ | 1.00 (reference) | 1.06 (0.97-1.15) | 1.00 (0.92-1.09) | 1.06 (0.97-1.16) | 0.382 |
| Model 2^c^ | 1.00 (reference) | 1.08 (0.99-1.18) | 1.04 (0.95-1.13) | 1.13 (1.04-1.25) | 0.017 |
| Model 3^d^ | 1.00 (reference) | 1.07 (0.97-1.18) | 1.02 (0.92-1.14) | 1.12 (0.98-1.27) | 0.187 |
| **TFAs, g. day^-1^.kcal^-1^** | <3.83 | 3.83-4.72 | 4.72-5.58 | >5.58 |  |
| Cases, n | 1096 | 1100 | 1060 | 1052 |  |
| Unadjusted | 1.00 (reference) | 1.01 (0.93-1.10) | 0.98 (0.90-1.07) | 0.98 (0.90-1.07) | 0.490 |
| Model 1^b^ | 1.00 (reference) | 1.00 (0.91-1.08) | 0.94 (0.87-1.03) | 0.95 (0.87-1.03) | 0.133 |
| Model 2^c^ | 1.00 (reference) | 0.99 (0.91-1.09) | 0.96 (0.88-1.06) | 1.00 (0.91-1.09) | 0.758 |
| Model 3^d^ | 1.00 (reference) | 0.96 (0.88-1.06) | 0.93 (0.84-1.03) | 0.95 (0.86-1.06) | 0.283 |
| ^a^ Values are hazard ratios (95% confidence intervals).  ^b^ Model 1: adjusted for age (years) and race (non-Hispanic White, non-Hispanic Black, Hispanic, and other race/ethnicity).  ^c^ Model 2: adjusted for mode 1 + smoking status [current (>20 cigarettes/day, 10-20 cigarettes/day, <10 cigarettes/day), former (stop smoking >15 years, stop smoking ≤15 years), never], alcohol consumption (g/day), body mass index (kg/m2), aspirin use (yes, no), history of diabetes (yes, no), family of prostate cancer (yes, no), energy intake from diet (kcal/day), and educational level.  ^d^ Model 3: adjusted for model 2 + protein(g/day), sodium (mg/day), carbohydrates (g/day), and dietary fiber (g/day). | | | | | |
|  | | | | | |

| **eTable 8.** Association between fat and specific fat type intake and the mortality of PCa ^a^. | | | | | |
| --- | --- | --- | --- | --- | --- |
|  | **Quartile 1** | **Quartile 2** | **Quartile 3** | **Quartile 4** | ***P-*trend** |
| **Total fat, g. day^-1^.kcal^-1^** | <54.29 | 54.29-62.46 | 62.46-70.95 | >70.95 |  |
| Cases, n | 91 | 99 | 101 | 101 |  |
| Unadjusted | 1.00 (reference) | 1.15 (0.85-1.53) | 1.17 (0.88-1.56) | 1.27 (0.96-1.68) | 0.108 |
| Model 1^b^ | 1.00 (reference) | 1.13 (0.85-1.51) | 1.21 (0.91-1.62) | 1.44 (1.08-1.91) | 0.011 |
| Model 2^c^ | 1.00 (reference) | 1.15 (0.86-1.56) | 1.25 (0.92-1.69) | 1.40 (1.03-1.90) | 0.027 |
| Model 3^d^ | 1.00 (reference) | 1.05 (0.75-1.48) | 1.09 (0.73-1.61) | 1.11 (0.65-1.88) | 0.696 |
| **MUFAs, g. day^-1^.kcal^-1^** | <20.12 | 20.12-23.59 | 23.59-26.92 | >26.92 |  |
| Cases, n | 87 | 97 | 107 | 101 |  |
| Unadjusted | 1.00 (reference) | 1.11 (0.84-1.48) | 1.15 (0.86-1.52) | 1.15 (0.87-1.53) | 0.320 |
| Model 1^b^ | 1.00 (reference) | 1.10 (0.83-1.46) | 1.20 (0.91-1.60) | 1.30 (0.98-1.73) | 0.056 |
| Model 2^c^ | 1.00 (reference) | 1.12 (0.83-1.51) | 1.23 (0.91-1.65) | 1.26 (0.93-1.70) | 0.112 |
| Model 3^d^ | 1.00 (reference) | 0.96 (0.69-1.34) | 0.97 (0.67-1.41) | 0.86(0.53-1.40) | 0.573 |
| **PUFAs, g. day^-1^.kcal^-1^** | <11.53 | 11.53-14.08 | 14.08-16.14 | >16.14 |  |
| Cases, n | 90 | 115 | 91 | 96 |  |
| Unadjusted | 1.00 (reference) | 0.80 (0.61-1.07) | 0.91 (0.70-1.20) | 0.93 (0.71-1.22) | 0.823 |
| Model 1^b^ | 1.00 (reference) | 0.79 (0.59-1.04) | 0.92 (0.70-1.22) | 1.00 (0.76-1.31) | 0.742 |
| Model 2^c^ | 1.00 (reference) | 0.82 (0.61-1.10) | 0.96 (0.73-1.28) | 1.01 (0.76-1.33) | 0.681 |
| Model 3^d^ | 1.00 (reference) | 0.73 (0.54-1.00) | 0.83 (0.61-1.13) | 0.79 (0.55-1.13) | 0.311 |
| **SFAs, g. day^-1^.kcal^-1^** | <16.47 | 16.47-19.92 | 19.92-23.03 | >23.03 |  |
| Cases, n | 89 | 100 | 103 | 100 |  |
| Unadjusted | 1.00 (reference) | 1.25 (0.93-1.68) | 1.38 (1.04-1.84) | 1.39 (1.04-1.86) | 0.018 |
| Model 1^b^ | 1.00 (reference) | 1.25 (0.93-1.67) | 1.44 (1.08-1.92) | 1.57 (1.17-2.11) | 0.001 |
| Model 2^c^ | 1.00 (reference) | 1.31 (0.96-1.78) | 1.49 (1.10-2.03) | 1.50 (1.10-2.05) | 0.008 |
| Model 3^d^ | 1.00 (reference) | 1.23 (0.88-1.72) | 1.37 (0.95-1.98) | 1.32 (0.85-2.06) | 0.192 |
| **TFAs, g. day^-1^.kcal^-1^** | <3.83 | 3.83-4.73 | 4.73-5.58 | >5.58 |  |
| Cases, n | 88 | 104 | 110 | 90 |  |
| Unadjusted | 1.00 (reference) | 1.03 (0.78-1.35) | 0.93 (0.70-1.24) | 1.10 (0.84-1.46) | 0.649 |
| Model 1^b^ | 1.00 (reference) | 0.99 (0.74-1.29) | 0.88 (0.66-1.18) | 1.11 (0.84-1.46) | 0.660 |
| Model 2^c^ | 1.00 (reference) | 1.00 (0.75-1.34) | 0.90 (0.66-1.22) | 1.10 (0.82-1.47) | 0.727 |
| Model 3^d^ | 1.00 (reference) | 0.91 (0.67-1.23) | 0.77 (0.56-1.08) | 0.92 (0.66-1.28) | 0.463 |
| ^a^ Values are hazard ratios (95% confidence intervals).  ^b^ Model 1: adjusted for age (years), sex (male, female), and race (non-Hispanic White, non-Hispanic Black, Hispanic, and other race/ethnicity).  ^c^ Model 2: adjusted for mode 1 + smoking status [current (>20 cigarettes/day, 10-20 cigarettes/day, <10 cigarettes/day), former (stop smoking >15 years, stop smoking ≤15 years), never], alcohol consumption (g/day), body mass index (kg/m2), aspirin use (yes, no), history of diabetes (yes, no), family of PCa (yes, no), energy intake from diet (kcal/day), and educational level.  ^d^ Model 3: adjusted for model 2 + protein(g/day), sodium (mg/day), carbohydrates (g/day), and dietary fiber (g/day). | | | | | |

| **eTable 9.** Subgroup analyses on the association between fat and SFAs from dairy and the incidence of PCa ^1^ | | | | |
| --- | --- | --- | --- | --- |
| Subgroup variable | Total fat from dairy intake and the incidence of PCa | | SFAs from dairy intake and the incidence of PCa | |
|  | Quartile 1 | Quartile 4 | Quartile 1 | Quartile 4 |
| **Age at diet history questionnaire completion (years)** | | | | |
| ≥65 | | | | |
| HR (95% CI) | 1.00 (reference) | 1.18 (1.03, 1.34) | 1.00 (reference) | 1.18 (1.03, 1.34) |
| *P* _for trend_ |  | 0.012 |  | 0.008 |
| <65 | | | | |
| HR (95% CI) | 1.00 (reference) | 1.08 (0.92, 1.26) | 1.00 (reference) | 1.05 (0.90, 1.23) |
| *P* _for trend_ |  | 0.623 |  | 0.604 |
| *P*_interaction_ |  | 0.418 |  | 0.271 |
| **Body mass index (kg/m^2^)** | | | | |
| ≥25 | | | | |
| HR (95% CI) | 1.00 (reference) | 1.08 (0.96, 1.22) | 1.00 (reference) | 1.07 (0.95, 1.21) |
| *P* _for trend_ |  | 0.226 |  | 0.153 |
| <25 | | | | |
| HR (95% CI) | 1.00 (reference) | 1.26 (1.03, 1.52) | 1.00 (reference) | 1.25 (1.03, 1.52) |
| *P* _for trend_ |  | 0.031 |  | 0.028 |
| *P*_interaction_ |  | 0.261 |  | 0.233 |
| **Smoking status** | | | | |
| Current or former smokers stopping smoking ≤15 years | | | | |
| HR (95% CI) | 1.00 (reference) | 1.18 (0.81, 1.71) | 1.00 (reference) | 1.24 (0.86, 1.80) |
| *P* _for trend_ |  | 0.657 |  | 0.613 |
| Never or former smokers stopping smoking >15 years | | | | |
| HR (95% CI) | 1.00 (reference) | 1.14 (1.03, 1.27) | 1.00 (reference) | 1.12 (1.01, 1.25) |
| *P* _for trend_ |  | 0.013 |  | 0.009 |
| *P*_interaction_ |  | 0.590 |  | 0.912 |
| **Alcohol consumption** | | | | |
| ≥ median | | | | |
| HR (95% CI) | 1.00 (reference) | 1.10 (0.98, 1.24) | 1.00 (reference) | 1.09 (0.97, 1.22) |
| *P* _for trend_ |  | 0.141 |  | 0.110 |
| < median |  |  |  |  |
| HR (95% CI) | 1.00 (reference) | 1.11 (0.93, 1.32) | 1.00 (reference) | 1.10 (0.93, 1.31) |
| *P* _for trend_ |  | 0.073 |  | 0.073 |
| *P*_interaction_ |  | 0.446 |  | 0.325 |
| **Aspirin use** | | | | |
| Yes | | | | |
| HR (95% CI) | 1.00 (reference) | 1.14 (0.99, 1.32) | 1.00 (reference) | 1.18 (1.03, 1.36) |
| *P* _for trend_ |  | 0.163 |  | 0.040 |
| No | | | | |
| HR (95% CI) | 1.00 (reference) | 1.11 (0.96, 1.29) | 1.00 (reference) | 1.06 (0.91, 1.22) |
| *P* _for trend_ |  | 0.088 |  | 0.178 |
| *P*_interaction_ |  | 0.362 |  | 0.368 |
| **History of diabetes** |  |  |  |  |
| Yes |  |  |  |  |
| HR (95% CI) | 1.00 (reference) | 1.45 (0.92, 2.27) | 1.00 (reference) | 1.43 (0.92, 2.23) |
| *P* _for trend_ |  | 0.103 |  | 0.101 |
| No |  |  |  |  |
| HR (95% CI) | 1.00 (reference) | 1.11 (1.00, 1.24) | 1.00 (reference) | 1.10 (0.99, 1.23) |
| *P* _for trend_ |  | 0.053 |  | 0.035 |
| *P*_interaction_ |  | 0.310 |  | 0.327 |
| **Trial group** | | | | |
| Screening group | | | | |
| HR (95% CI) | 1.00 (reference) | 1.12 (0.97, 1.30) | 1.00 (reference) | 1.13 (0.98, 1.30) |
| *P* _for trend_ |  | 0.179 |  | 0.110 |
| Control group | | | | |
| HR (95% CI) | 1.00 (reference) | 1.14 (0.98, 1.32) | 1.00 (reference) | 1.12 (0.97, 1.29) |
| *P* _for trend_ |  | 0.064 |  | 0.062 |
| *P*_interaction_ |  | 0.812 |  | 0.981 |

^1^ n = 49,424. Hazard ratios and 95% confidence intervals were calculated using the Cox proportional hazards regression model. HRs were adjusted for age (years), sex (male, female), and race (non-Hispanic White, non-Hispanic Black, Hispanic, and other race/ethnicity), smoking status [current (>20 cigarettes/day, 10-20 cigarettes/day, <10 cigarettes/day), former (stop smoking >15 years, stop smoking ≤15 years), never], alcohol consumption (g/day), body mass index (kg/m2), aspirin use (yes, no), history of diabetes (yes, no), family of PCa (yes, no), energy intake from diet (kcal/day), educational level, protein(g/day), sodium (mg/day), carbohydrates (g/day), and dietary fiber (g/day). In subgroup analyses stratified by age, body mass index, smoking status, alcohol consumption, aspirin use, history of diabetes and trial group, hazard ratios were not adjusted for the stratification factor. The *P*_interaction_ was calculated by comparing models with and without interaction terms. CI, confidence interval; HR, hazard ratio.

| **eTable 10.** Subgroup analyses on the association between MUFAs and SFAs from plant PUFAs from fish and the mortality of PCa ^1^ | | | | | | |
| --- | --- | --- | --- | --- | --- | --- |
| Subgroup variable | MUFAs from plant intake and the mortality of prostate cancer | | PUFAs from fish intake and the morality of prostate cancer | | SFAs from plant intake and the mortality of prostate cancer | |
|  | Quartile 1 | Quartile 4 | Quartile 1 | Quartile 4 | Quartile 1 | Quartile 4 |
| **Age at diet history questionnaire completion (years)** | | | | | | |
| ≥65 | | | | | | |
| HR (95% CI) | 1.00 (reference) | 0.79 (0.53, 1.17) | 1.00 (reference) | 0.54 (0.38, 0.78) | 1.00 (reference) | 0.66 (0.44, 0.97) |
| *P* _for trend_ |  | 0.779 |  | 0.002 |  | 0.358 |
| <65 | | |  |  |  |  |
| HR (95% CI) | 1.00 (reference) | 0.45 (0.24, 0.84) | 1.00 (reference) | 0.90 (0.52, 1.54) | 1.00 (reference) | 0.51 (0.27, 0.94) |
| *P* _for trend_ |  | 0.036 |  | 0.780 |  | 0.083 |
| *P*_interaction_ |  | 0.230 |  | **0.048** |  | 0.562 |
| **Body mass index (kg/m^2^)** | | | | | | |
| ≥25 | | | | | | |
| HR (95% CI) | 1.00 (reference) | 0.56 (0.38, 0.82) | 1.00 (reference) | 0.57 (0.41, 0.80) | 1.00 (reference) | 0.62 (0.42, 0.92) |
| *P* _for trend_ |  | 0.067 |  | 0.002 |  | 0.128 |
| <25 | | |  | |  | |
| HR (95% CI) | 1.00 (reference) | 1.10 (0.56, 2.14) | 1.00 (reference) | 0.97 (0.51, 1.83) | 1.00 (reference) | 0.71 (0.37, 1.39) |
| *P* _for trend_ |  | 0.755 |  | 0.296 |  | 0.813 |
| *P*_interaction_ |  | 0.326 |  | 0.056 |  | 0.694 |
| **Smoking status** | | |  |  |  |  |
| Current or former smokers stopping smoking ≤15 years | | |  |  |  |  |
| HR (95% CI) | 1.00 (reference) | 0.48 (0.17, 1.36) | 1.00 (reference) | 0.92 (0.34, 2.48) | 1.00 (reference) | 0.62 (0.24, 1.65) |
| *P* _for trend_ |  | 0.061 |  | 0.740 |  | 0.257 |
| Never or former smokers stopping smoking >15 years | | |  |  |  |  |
| HR (95% CI) | 1.00 (reference) | 0.69 (0.48, 0.98) | 1.00 (reference) | 0.64 (0.47, 0.88) | 1.00 (reference) | 0.64 (0.45, 0.92) |
| *P* _for trend_ |  | 0.432 |  | 0.021 |  | 0.279 |
| *P*_interaction_ |  | 0.162 |  | 0.277 |  | 0.373 |
| **Alcohol consumption** | | |  |  |  |  |
| ≥ median | | |  |  |  |  |
| HR (95% CI) | 1.00 (reference) | 0.71 (0.49, 1.02) | 1.00 (reference) | 0.83 (0.57, 1.23) | 1.00 (reference) | 0.71 (0.50, 1.02) |
| *P* _for trend_ |  | 0.234 |  | 0.818 |  | 0.226 |
| < median |  |  |  |  |  |  |
| HR (95% CI) | 1.00 (reference) | 0.72 (0.42, 1.24) | 1.00 (reference) | 0.50 (0.30, 0.82) | 1.00 (reference) | 0.67 (0.38, 1.17) |
| *P* _for trend_ |  | 0.605 |  | 0.004 |  | 0.602 |
| *P*_interaction_ |  | 0.518 |  | **0.037** |  | 0.875 |
| **Aspirin use** | | |  |  |  |  |
| Yes | | |  |  |  |  |
| HR (95% CI) | 1.00 (reference) | 0.61 (0.38, 0.97) | 1.00 (reference) | 0.52 (0.34, 0.79) | 1.00 (reference) | 0.63 (0.39, 1.02) |
| *P* _for trend_ |  | 0.129 |  | 0.005 |  | 0.215 |
| No | | |  |  |  |  |
| HR (95% CI) | 1.00 (reference) | 0.76 (0.48, 1.21) | 1.00 (reference) | 0.83 (0.55, 1.25) | 1.00 (reference) | 0.67 (0.42, 1.06) |
| *P* _for trend_ |  | 0.643 |  | 0.798 |  | 0.395 |
| *P*_interaction_ |  | 0.777 |  | 0.519 |  | 0.826 |
| **History of diabetes** |  |  |  |  |  |  |
| Yes |  |  |  |  |  |  |
| HR (95% CI) | 1.00 (reference) | 0.24 (0.35, 1.67) | 1.00 (reference) | 0.60 (0.14, 2.60) | 1.00 (reference) | 0.20 (0.03, 1.42) |
| *P* _for trend_ |  | 0.036 |  | 0.606 |  | 0.021 |
| No |  |  |  |  |  |  |
| HR (95% CI) | 1.00 (reference) | 0.70 (0.45, 0.98) | 1.00 (reference) | 0.65 (0.48, 0.88) | 1.00 (reference) | 0.68 (0.48, 0.95) |
| *P* _for trend_ |  | 0.337 |  | 0.031 |  | 0.322 |
| *P*_interaction_ |  | 0.085 |  | 0.650 |  | 0.081 |
| **Trial group** | | |  |  |  |  |
| Screening group | | |  |  |  |  |
| HR (95% CI) | 1.00 (reference) | 0.84 (0.52, 1.36) | 1.00 (reference) | 0.74 (0.48, 1.13) | 1.00 (reference) | 0.76 (0.47, 1.21) |
| *P* _for trend_ |  | 0.593 |  | 0.135 |  | 0.359 |
| Control group | | |  |  |  |  |
| HR (95% CI) | 1.00 (reference) | 0.55 (0.35, 0.88) | 1.00 (reference) | 0.59 (0.39, 0.89) | 1.00 (reference) | 0.56 (0.35, 0.90) |
| *P* _for trend_ |  | 0.851 |  | 0.021 |  | 0.026 |
| *P*_interaction_ |  | 0.307 |  | 0.304 |  | 0.384 |

^1^ n = 49,424. Hazard ratios and 95% confidence intervals were calculated using the Cox proportional hazards regression model. HRs were adjusted for age (years), sex (male, female), and race (non-Hispanic White, non-Hispanic Black, Hispanic, and other race/ethnicity), smoking status [current (>20 cigarettes/day, 10-20 cigarettes/day, <10 cigarettes/day), former (stop smoking >15 years, stop smoking ≤15 years), never], alcohol consumption (g/day), body mass index (kg/m2), aspirin use (yes, no), history of diabetes (yes, no), family of PCa (yes, no), energy intake from diet (kcal/day), educational level, protein(g/day), sodium (mg/day), carbohydrates (g/day), and dietary fiber (g/day). In subgroup analyses stratified by age, body mass index, smoking status, alcohol consumption, aspirin use, history of diabetes and trial group, hazard ratios were not adjusted for the stratification factor. The *P*_interaction_ was calculated by comparing models with and without interaction terms. CI, confidence interval; HR, hazard ratio.

| **eTable 11.** Sensitivity analyses on the association between total fat from dairy and the incidence of PCa a | | | | | | |
| --- | --- | --- | --- | --- | --- | --- |
| Categories | Sample size | Quartiles of energy-adjusted total fat from dairy | | | | *P-*trend |
|  |  | Quartile 1 | Quartile 2 | Quartile 3 | Quartile 4 |  |
| Excluded subjects with extreme  BMIs b | 46990 | 1.00 (reference) | 1.05 (0.95, 1.15) | 0.98 (0.89, 1.09) | 1.13 (1.02, 1.26) | 0.069 |
| Excluded cases occurred within the first two years of follow-up | 49330 | 1.00 (reference) | 1.04 (0.94, 1.14) | 0.97 (0.88, 1.08) | 1.13 (1.02, 1.25) | 0.067 |
| Excluded cases occurred within the first three years of follow-up | 49175 | 1.00 (reference) | 1.03 (0.94, 1.13) | 0.98 (0.88, 1.08) | 1.14 (1.03, 1.26) | 0.048 |
| Excluded cases occurred within the first five years of follow-up | 48014 | 1.00 (reference) | 1.05 (0.95, 1.16) | 1.00 (0.90, 1.12) | 1.16 (1.03, 1.29) | 0.035 |
| Repeated the analysis with complete covariate data | 47772 | 1.00 (reference) | 1.03 (0.94, 1.13) | 0.97 (0.88, 1.07) | 1.12 (1.01, 1.24) | 0.100 |
| Additional adjustment on model 3 |  |  |  |  |  |  |
| Healthy Eating Index-2015 and Physical activity | 49424 | 1.00 (reference) | 1.04 (0.94, 1.14) | 0.99 (0.89, 1.09) | 1.16 (1.05, 1.30) | 0.021 |
| Physical activity and intakes of fruit,vegetable and whole grain | 49424 | 1.00 (reference) | 1.04 (0.94, 1.14) | 0.98 (0.88, 1.08) | 1.13 (1.02, 1.26) | 0.061 |
| Physical activity and intakes of , processed and unprocessed red and white meat | 49424 | 1.00 (reference) | 1.03 (0.94, 1.14) | 0.98 (0.89, 1.08) | 1.15 (1.04, 1.29) | 0.031 |

a Values are hazard ratios (95% confidence intervals). Hazard ratios were adjusted for the following variables unless otherwise specified: age (years), and race (non-Hispanic White, non-Hispanic Black, Hispanic, and other race/ethnicity), smoking status [current (>20 cigarettes/day, 10-20 cigarettes/day, <10 cigarettes/day), former (stop smoking >15 years, stop smoking ≤15 years), never], alcohol consumption (g/day), body mass index (kg/m2), aspirin use (yes, no), history of diabetes , family of prostate cancer (yes, no), energy intake from diet (kcal/day), and educational level, protein(g/day), sodium (mg/day), carbohydrates (g/day), and dietary fiber (g/day).

b Participants who had extreme BMIs (top 2.5% or bottom 2.5% of BMI ).

| **eTable 12.** Sensitivity analyses on the association between SFAs from dairy and the incidence of PCa a | | | | | | |
| --- | --- | --- | --- | --- | --- | --- |
| Categories | Sample size | Quartiles of energy-adjusted SFA from dairy | | | | *P* trend |
|  |  | Quartile 1 | Quartile 2 | Quartile 3 | Quartile 4 |  |
| Excluded subjects with extreme  BMIs b | 46990 | 1.00 (reference) | 1.03 (0.94, 1.14) | 1.01 (0.91, 1.11) | 1.13 (1.02, 1.25) | 0.046 |
| Excluded cases occurred within the first two years of follow-up | 49330 | 1.00 (reference) | 1.02 (0.93, 1.12) | 0.99 (0.90, 1.09) | 1.12 (1.01, 1.24) | 0.053 |
| Excluded cases occurred within the first three years of follow-up | 49175 | 1.00 (reference) | 1.02 (0.92, 1.12) | 1.00 (0.90, 1.10) | 1.13 (1.02, 1.26) | 0.031 |
| Excluded cases occurred within the first five years of follow-up | 48014 | 1.00 (reference) | 1.02 (0.92, 1.13) | 1.00 (0.90, 1.11) | 1.15 (1.03, 1.28) | 0.029 |
| Repeated the analysis with complete covariate data | 47772 | 1.00 (reference) | 1.02 (0.93, 1.12) | 0.99 (0.90, 1.09) | 1.11 (1.00, 1.23) | 0.087 |
| Additional adjustment on model 3 c |  |  |  |  |  |  |
| Healthy Eating Index-2015 and Physical activity | 49424 | 1.00 (reference) | 1.02 (0.93, 1.12) | 1.00 (0.91, 1.11) | 1.15 (1.04, 1.28) | 0.019 |
| Physical activity and intakes of fruit,vegetable, red and white meat and whole grain | 49424 | 1.00 (reference) | 1.02 (0.93, 1.12) | 1.00 (0.90, 1.10) | 1.12 (1.01, 1.24) | 0.057 |
| Physical activity and intakes of protein, fat, sodium, carbohydrates, and dietary fiber | 49424 | 1.00 (reference) | 1.03 (0.93, 1.13) | 1.00 (0.91, 1.11) | 1.15 (1.03, 1.28) | 0.024 |

a Values are hazard ratios (95% confidence intervals). Hazard ratios were adjusted for the following variables unless otherwise specified: age (years), and race (non-Hispanic White, non-Hispanic Black, Hispanic, and other race/ethnicity), smoking status [current (>20 cigarettes/day, 10-20 cigarettes/day, <10 cigarettes/day), former (stop smoking >15 years, stop smoking ≤15 years), never], alcohol consumption (g/day), body mass index (kg/m2), aspirin use (yes, no), history of diabetes , family of prostate cancer (yes, no), energy intake from diet (kcal/day), and educational level, protein(g/day), sodium (mg/day), carbohydrates (g/day), and dietary fiber (g/day).

b Participants who had extreme BMIs (top 2.5% or bottom 2.5% of BMI ).

| **eTable 13.** Sensitivity analyses on the association between MUFAs from plants and the mortality of PCa a | | | | | | |
| --- | --- | --- | --- | --- | --- | --- |
| Categories | Sample size | Quartiles of energy-adjusted MUFAs from plants | | | | *P* trend |
|  |  | Quartile 1 | Quartile 2 | Quartile 3 | Quartile 4 |  |
| Excluded subjects with extreme  BMIs b | 46990 | 1.00 (reference) | 0.72 (0.53, 0.97) | 0.69 (0.51, 0.94) | 0.66 (0.47, 0.93) | 0.021 |
| Excluded cases occurred within the first two years of follow-up | 49330 | 1.00 (reference) | 0.75 (0.56, 1.01) | 0.71 (0.52, 0.96) | 0.67 (0.48, 0.94) | 0.022 |
| Excluded cases occurred within the first three years of follow-up | 49175 | 1.00 (reference) | 0.75 (0.56, 1.00) | 0.68 (0.50, 0.92) | 0.66 (0.47, 0.92) | 0.013 |
| Excluded cases occurred within the first five years of follow-up | 48014 | 1.00 (reference) | 0.70 (0.51, 0.95) | 0.66 (0.48, 0.91) | 0.68 (0.48, 0.96) | 0.034 |
| Repeated the analysis with complete covariate data | 47772 | 1.00 (reference) | 0.72 (0.54, 0.97) | 0.71 (0.52, 0.97) | 0.66 (0.47, 0.93) | 0.023 |
| Additional adjustment on model 3 |  |  |  |  |  |  |
| Healthy Eating Index-2015 and Physical activity | 49424 | 1.00 (reference) | 0.74 (0.55, 1.00) | 0.70 (0.51, 0.96) | 0.67 (0.47, 0.95) | 0.029 |
| Physical activity and intakes of fruit,vegetable and whole grain | 49424 | 1.00 (reference) | 0.75 (0.56, 1.00) | 0.71 (0.52, 0.96) | 0.68 (0.48, 0.94) | 0.024 |
| Physical activity and intakes of protein, fat, sodium, carbohydrates, and dietary fiber | 49424 | 1.00 (reference) | 0.75 (0.56, 1.01) | 0.71 (0.53, 0.97) | 0.69 (0.49, 0.96) | 0.033 |

a Values are hazard ratios (95% confidence intervals). Hazard ratios were adjusted for the following variables unless otherwise specified: age (years), and race (non-Hispanic White, non-Hispanic Black, Hispanic, and other race/ethnicity), smoking status [current (>20 cigarettes/day, 10-20 cigarettes/day, <10 cigarettes/day), former (stop smoking >15 years, stop smoking ≤15 years), never], alcohol consumption (g/day), body mass index (kg/m2), aspirin use (yes, no), history of diabetes , family of prostate cancer (yes, no), energy intake from diet (kcal/day), and educational level, protein(g/day), sodium (mg/day), carbohydrates (g/day), and dietary fiber (g/day).

b Participants who had extreme BMIs (top 2.5% or bottom 2.5% of BMI ).

| **eTable 14.** Sensitivity analyses on the association between PUFAs from plants and the mortality of PCa a | | | | | | |
| --- | --- | --- | --- | --- | --- | --- |
| Categories | Sample size | Quartiles of energy-adjusted PUFA from fish | | | | *P* trend |
|  |  | Quartile 1 | Quartile 2 | Quartile 3 | Quartile 4 |  |
| Excluded subjects with extreme  BMIs b | 46990 | 1.00 (reference) | 0.58 (0.44, 0.77) | 0.54 (0.40, 0.72) | 0.62 (0.45, 0.83) | 0.002 |
| Excluded cases occurred within the first two years of follow-up | 49330 | 1.00 (reference) | 0.59 (0.44, 0.78) | 0.56 (0.42, 0.75) | 0.65 (0.48, 0.87) | 0.006 |
| Excluded cases occurred within the first three years of follow-up | 49175 | 1.00 (reference) | 0.57 (0.42, 0.75) | 0.55 (0.41, 0.74) | 0.65 (0.48, 0.87) | 0.007 |
| Excluded cases occurred within the first five years of follow-up | 48014 | 1.00 (reference) | 0.57 (0.42, 0.78) | 0.59 (0.43, 0.79) | 0.69 (0.51, 0.95) | 0.037 |
| Repeated the analysis with complete covariate data | 47772 | 1.00 (reference) | 0.58 (0.43, 0.77) | 0.55 (0.41, 0.73) | 0.63 (0.47, 0.85) | 0.004 |
| Additional adjustment on model 3 |  |  |  |  |  |  |
| Healthy Eating Index-2015 and Physical activity | 49424 | 1.00 (reference) | 0.59 (0.45, 0.78) | 0.56 (0.42, 0.74) | 0.65 (0.48, 0.88) | 0.006 |
| Physical activity and intakes of fruit,vegetable, red and white meat and whole grain | 49424 | 1.00 (reference) | 0.59 (0.45, 0.78) | 0.56 (0.42, 0.74) | 0.65 (0.48, 0.87) | 0.005 |
| Physical activity and intakes of protein, fat, sodium, carbohydrates, and dietary fiber | 49424 | 1.00 (reference) | 0.59 (0.45, 0.79) | 0.56 (0.42, 0.75) | 0.67 (0.49, 0.91) | 0.014 |

a Values are hazard ratios (95% confidence intervals). Hazard ratios were adjusted for the following variables unless otherwise specified: age (years), and race (non-Hispanic White, non-Hispanic Black, Hispanic, and other race/ethnicity), smoking status [current (>20 cigarettes/day, 10-20 cigarettes/day, <10 cigarettes/day), former (stop smoking >15 years, stop smoking ≤15 years), never], alcohol consumption (g/day), body mass index (kg/m2), aspirin use (yes, no), history of diabetes , family of prostate cancer (yes, no), energy intake from diet (kcal/day), and educational level, protein(g/day), sodium (mg/day), carbohydrates (g/day), and dietary fiber (g/day).

b Participants who had extreme BMIs (top 2.5% or bottom 2.5% of BMI ).

| **eTable 15.** Sensitivity analyses on the association between SFAs from plants and the mortality of PCa a | | | | | | |
| --- | --- | --- | --- | --- | --- | --- |
| Categories | Sample size | Quartiles of energy-adjusted SFAs from plant | | | | *P* trend |
|  |  | Quartile 1 | Quartile 2 | Quartile 3 | Quartile 4 |  |
| Excluded subjects with extreme  BMIs b | 46990 | 1.00 (reference) | 0.67 (0.50, 0.91) | 0.71 (0.52, 0.96) | 0.67 (0.48, 0.95) | 0.041 |
| Excluded cases occurred within the first two years of follow-up | 49330 | 1.00 (reference) | 0.67 (0.50, 0.89) | 0.72 (0.53, 0.96) | 0.65 (0.46, 0.91) | 0.025 |
| Excluded cases occurred within the first three years of follow-up | 49175 | 1.00 (reference) | 0.65 (0.49, 0.88) | 0.70 (0.52, 0.94) | 0.63 (0.45, 0.89) | 0.018 |
| Excluded cases occurred within the first five years of follow-up | 48014 | 1.00 (reference) | 0.70 (0.51, 0.95) | 0.72 (0.52, 0.99) | 0.66 (0.46, 0.95) | 0.040 |
| Repeated the analysis with complete covariate data | 47772 | 1.00 (reference) | 0.63 (0.47, 0.85) | 0.70 (0.52, 0.95) | 0.62 (0.45, 0.88) | 0.020 |
| Additional adjustment on model 3 c |  |  |  |  |  |  |
| Healthy Eating Index-2015 and Physical activity | 49424 | 1.00 (reference) | 0.66 (0.49, 0.89) | 0.72 (0.53, 0.97) | 0.65 (0.46, 0.92) | 0.031 |
| Physical activity and intakes of fruit,vegetable, red and white meat and whole grain | 49424 | 1.00 (reference) | 0.66 (0.49, 0.89) | 0.72 (0.53, 0.97) | 0.65 (0.47, 0.91) | 0.028 |
| Physical activity and intakes of protein, fat, sodium, carbohydrates, and dietary fiber | 49424 | 1.00 (reference) | 0.66 (0.49, 0.89) | 0.73 (0.54, 0.98) | 0.66 (0.47, 0.93) | 0.036 |

a Values are hazard ratios (95% confidence intervals). Hazard ratios were adjusted for the following variables unless otherwise specified: age (years), and race (non-Hispanic White, non-Hispanic Black, Hispanic, and other race/ethnicity), smoking status [current (>20 cigarettes/day, 10-20 cigarettes/day, <10 cigarettes/day), former (stop smoking >15 years, stop smoking ≤15 years), never], alcohol consumption (g/day), body mass index (kg/m2), aspirin use (yes, no), history of diabetes , family of prostate cancer (yes, no), energy intake from diet (kcal/day), and educational level, protein(g/day), sodium (mg/day), carbohydrates (g/day), and dietary fiber (g/day).

b Participants who had extreme BMIs (top 2.5% or bottom 2.5% of BMI ).
